# Recent Advances in Enzymatic Biofuel Cells to Power Up Wearable and Implantable Biosensors

**DOI:** 10.3390/bios15040218

**Published:** 2025-03-28

**Authors:** Zina Fredj, Guoguang Rong, Mohamad Sawan

**Affiliations:** CenBRAIN Neurotech, School of Engineering, Westlake University, Hangzhou 310030, China; zinafraj@westlake.edu.cn (Z.F.); rongguoguang@westlake.edu.cn (G.R.)

**Keywords:** enzymatic biofuel cells, energy sources, self-powered biosensors, wearable devices, implantable sensors, remote sensing

## Abstract

Enzymatic biofuel cells (EBFCs) have emerged as a transformative solution in the quest for sustainable energy, offering a biocatalyst-driven alternative for powering wearable and implantable self-powered biosensors. These systems harness renewable enzyme activity under mild conditions, positioning them as ideal candidates for next-generation biosensing applications. Despite their promise, their practical deployment is limited by challenges such as low power density, restricted operational lifespan, and miniaturization complexities. This review provides an in-depth exploration of the evolving landscape of EBFC technology, beginning with fundamental principles and the latest developments in electron transfer mechanisms. A critical assessment of enzyme immobilization techniques, including physical adsorption, covalent binding, entrapment, and cross-linking, underscores the importance of optimizing enzyme stability and catalytic activity for enhanced bioelectrode performance. Additionally, we examine advanced bioelectrode materials, focusing on the role of nanostructures such as carbon-based nanomaterials, noble metals, conducting polymers, and metal–organic frameworks in improving electron transfer and boosting biosensor efficiency. Also, this review includes case studies of EBFCs in wearable self-powered biosensors, with particular attention to the real-time monitoring of neurotransmitters, glucose, lactate, and ethanol through sweat analysis, as well as their integration into implantable devices for continuous healthcare monitoring. Moreover, a dedicated discussion on challenges and trends highlights key limitations, including durability, power management, and scalability, while presenting innovative approaches to address these barriers. By addressing both technical and biological constraints, EBFCs hold the potential to revolutionize biomedical diagnostics and environmental monitoring, paving the way for highly efficient, autonomous biosensing platforms.

## 1. Introduction

Energy harvesting, capturing and storing energy from various environmental sources, has become essential in developing next-generation electronic devices. The capability to efficiently convert ambient energy into electrical power is particularly valuable for applications requiring autonomy and sustainability, such as wearable electronics, implantable medical devices, and remote sensors. Among the various energy harvesting techniques, biological fuel cells (BFCs) represent a promising approach that leverages biological processes to generate electricity under mild conditions [1,2].

Within the realm of BFCs, EBFCs have garnered considerable interest due to their unique capability to utilize enzymes as biocatalysts for energy conversion. Introduced by Yahiro et al., EBFCs oxidize substrates like sugars, alcohols, and organic acids at the anode while reducing oxidants such as dioxygen or peroxide at the cathode to produce water [3,4,5]. This process mimics natural metabolic pathways, allowing EBFCs to function efficiently in biologically relevant environments. Using oxidoreductases as biocatalysts in EBFCs offers several benefits, including excellent biocompatibility, high catalytic activity and specificity, and the capability to function under mild conditions [6]. These attributes make EBFCs ideal for miniaturization, supporting the development of wearable and implantable devices, as well as future applications that integrate with the human body. These applications could include self-powered sensors, therapeutic systems, and human–device interfaces [7,8,9]. While EBFCs offer significant advantages over traditional fuel cells, significant technical challenges currently limit their practical application, including low power output, limited operational stability, and the complex interactions between enzymes and electrode materials [10,11]. Our interest in BFCs stems from their potential as an energy source for self-powered biosensors (SPBs), a rapidly growing field with diverse applications, including continuous health monitoring, environmental sensing, and wearable medical devices. SPBs function with a simple two-electrode configuration, removing the reliance on an external power source [12,13]. Powered by biological fluids, these devices are particularly well suited for applications in implantable sensors. Katz et al. first introduced the concept of SPBs as a potentiometric system capable of detecting glucose and lactate [14]. Since then, much of the research has focused on the amperometric detection of glucose. Despite the clear advantages of EBFCs over conventional fuel cells, significant challenges hinder their seamless integration into biosensing platforms [15,16]. A key challenge arises from the inherent complexity of EBFCs, particularly the interactions among redox potentials of cofactors, mediators, and enzymes [17]. This complexity often results in an open circuit potential lower than theoretically predicted, adding an additional layer of difficulty compared to traditional fuel cells. Another critical challenge is maintaining enzyme stability and activity over extended operational periods, as enzymes often denature or lose functionality under non-ideal environmental conditions. Furthermore, the chemistry and surface characteristics of electrodes are crucial, as they directly influence the efficiency of electron transfer and enzyme stability by providing an optimal microenvironment that preserves enzyme structure and function [18,19]. Recent advancements in synthesizing and characterizing nanostructured materials have opened new opportunities to overcome these limitations [20,21]. Nanostructured electrodes, with their tailored composition and high surface area, significantly enhance enzyme immobilization efficiency and electron transfer kinetics. Additionally, innovative strategies such as enzyme encapsulation, covalent immobilization, and the use of conductive polymers or composites have shown promise in extending operational lifetimes and maintaining enzyme activity under physiological conditions. Continued development in these areas is essential for advancing EBFC technologies toward practical integration into wearable and implantable SPBs. This review discusses recent progress in EBFC technology and its significant potential for advancing wearable and implantable SPBs. It examines the fundamental principles underpinning EBFCs, delves into innovative approaches for enzyme immobilization, and evaluates the impact of nanostructured electrode materials on their performance. By addressing both technical and practical aspects, the paper provides a thorough analysis of the advantages, limitations, and optimization strategies for EBFC-based SPBs. Furthermore, this review investigates a broad range of applications, such as neurotransmitter detection, glucose sensing, and other emerging biomedical use cases, showcasing the versatility of these technologies. Through a detailed evaluation of recent advancements, critical technical challenges, and opportunities for improvement, this paper underscores the transformative role of EBFCs in enabling sustainable, self-powered biosensing platforms. Particular emphasis is placed on their potential integration with other energy harvesting and storage systems to enhance their efficiency and operational lifetime. Ultimately, this work aims to provide a roadmap for future research, highlighting the pivotal role of EBFCs in bridging the gap between sustainable energy generation and advanced biosensing applications.

## 2. Enzymatic Biofuel Cells

### 2.1. EBFC Fundamentals and Directions

**Figure 1:**
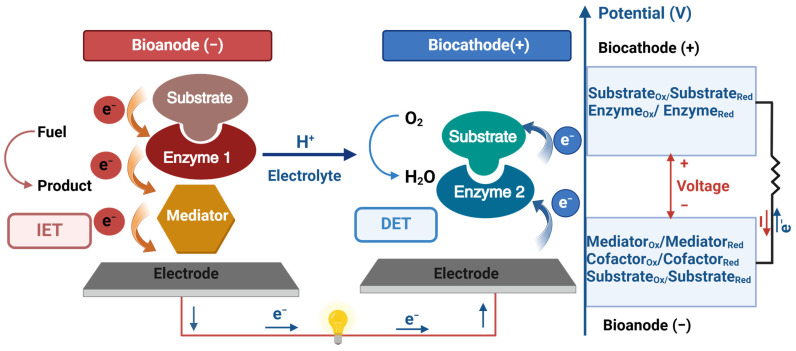
Schematic representation of an EBFC, demonstrating mediator-assisted electron transfer at the bioanode (−) and direct electron transfer at the biocathode (+). The diagram also highlights proton (H^+^) movement across the electrolyte and the generation of voltage within the system.

EBFCs leverage enzymatic catalysis at their core to oxidize or reduce fuel molecules, harnessing the energy released in these reactions to produce electrical power. Figure 1 presents a diagram of a typical enzymatic biofuel cell, detailing its essential components and functional mechanisms. The EBFC utilizes a mediated bioanode and a biocathode that relies on direct electron transfer. The mediated bioanode is responsible for the oxidation of the fuel, and it employs enzyme biocatalysts along with mediators to facilitate the electron movement from the fuel to the electrode interface. This process enables efficient energy conversion in the bioanode. Using a direct electron transfer mechanism, the biocathode is designed to reduce the electron acceptor, typically oxygen. This eliminates the need for mediators and simplifies the cathodic reaction, enhancing the overall efficiency of the EBFC.

Combining these components allows the EBFC to generate electrical energy through the enzymatic reactions occurring at the bioanode and biocathode. This sustainable energy source operates under mild conditions and harnesses the power of renewable enzyme biocatalysts and fuels [22]. One of the most significant advantages of EBFCs lies in the specificity of enzymes, which minimizes interference between catalytic processes [23]. This characteristic facilitates the design of EBFCs as single-chamber cells without separators, simplifying their construction and enhancing mass-transfer rates due to ambient convection [24]. Additionally, the power output of EBFCs is directly proportional to the concentration of the corresponding fuel, providing a scalable and efficient energy generation mechanism. As research progresses, the potential of EBFCs continues to expand, driven by innovations in enzyme engineering, electrode materials, and system integration. Nanotechnology holds particular promise in enhancing EBFC performance by developing novel electrode materials and enzyme immobilization techniques. Furthermore, advancements in bioengineering enable the customization of enzymes to optimize their catalytic activity and stability within EBFCs, paving the way for improved efficiency and durability [25,26]. Looking ahead, the evolving directions of EBFC research encompass a broad spectrum of applications, from portable electronics to biomedical devices. EBFCs are increasingly being explored as sustainable power sources for implantable medical devices, such as pacemakers and neurostimulators, offering an energy alternative to traditional battery technologies [27,28,29]. Additionally, integrating EBFCs into smart drug delivery systems holds the potential for precise and controlled release of therapeutic agents, revolutionizing personalized medicine [30,31].

### 2.2. Electron Transfer Mechanisms

Enzyme-based biofuel cell biosensors harness intricate electron transfer mechanisms pivotal in detecting and quantifying target substances. These biosensors rely on enzymatic catalysis, converting specific analytes into measurable signals via electron transfer processes. Two primary mechanisms govern electron transfer within these biosensors: Direct Electron transfer (DET) and indirect electron transfer (IET) [32]. In DET, electrons are transferred directly between the enzyme’s redox center and the electrode surface without the use of mediators. This mechanism is advantageous due to its simplicity, as it eliminates the need for additional chemical components, reducing potential interference and ensuring a rapid response time. DET-based biosensors often exhibit high specificity and improved long-term stability since the absence of mediators minimizes leaching effects. However, a key limitation of DET is its dependency on the spatial arrangement of enzymes. Many redox-active centers in enzymes, such as glucose oxidase (GOx), are buried within the protein structure, restricting efficient electron transfer unless the enzyme is positioned very close to the electrode. This poses a challenge in practical biosensor design, as it necessitates precise enzyme immobilization strategies. To address this, researchers have employed nanomaterials like carbon nanotubes and graphene, which enhance DET by facilitating closer enzyme-electrode contact and improving conductivity, as noted by Oh et al. [33]. Additionally, despite its advantages, DET generally provides weaker current outputs compared to IET, making it less suitable for applications requiring high sensitivity and signal amplification.

Conversely, IET employs mediators, small molecules, or cofactors that shuttle electrons between the enzyme and the electrode. This mechanism overcomes the spatial limitations of DET, as mediators can efficiently access deeply buried redox centers and enhance electron transfer kinetics. Common mediators used in EBFCs include ferrocene and methylene blue, which improve signal strength and allow biosensors to operate effectively even at low analyte concentrations. IET-based systems are particularly beneficial for biosensors utilizing oxidases, where direct interaction with the electrode is limited [34]. Furthermore, IET enhances flexibility in sensor design by allowing the use of a broader range of enzymes that might not naturally support DET. However, the main drawbacks of IET include potential mediator leakage, which can lead to signal instability, and the additional complexity of mediator selection, which must balance factors such as redox potential, solubility, and biocompatibility. Moreover, the use of synthetic mediators introduces potential toxicity concerns in biomedical applications, necessitating further optimization of mediator design for in vivo use.

Conversely, IET employs mediators, such as small molecules or cofactors, to shuttle electrons between the enzyme and the electrode.

This mechanism overcomes the spatial limitations of DET, as mediators can efficiently access deeply buried redox centers and enhance electron transfer kinetics. Common mediators used in EBFCs include ferrocene and methylene blue, which improve signal strength and allow biosensors to operate effectively even at low analyte concentrations. IET-based systems are particularly beneficial for biosensors utilizing oxidases, where direct interaction with the electrode is limited [34]. Furthermore, IET enhances flexibility in sensor design by allowing the use of a broader range of enzymes that might not naturally support DET. However, the main drawbacks of IET include potential mediator leakage, which can lead to signal instability, and the additional complexity of mediator selection, which must balance factors such as redox potential, solubility, and biocompatibility. Moreover, the use of synthetic mediators introduces potential toxicity concerns in biomedical applications, necessitating further optimization of mediator design for in vivo use. The selection between DET and IET depends on multiple factors, such as the nature of the enzyme, the properties of the analyte, and the design of the electrode surface. For instance, laccase enzymes are known to naturally support DET due to the accessibility of their redox centers. In contrast, many oxidase-based biosensors opt for IET, as mediators can enhance the reaction kinetics and sensitivity, especially when direct enzyme–electrode interaction is limited [35]. By acting as intermediaries, mediators improve both electron transfer rates and the versatility of biosensors, enabling the detection of a wider range of analytes. Recent developments in nanotechnology have greatly improved both DET and IET mechanisms in enzyme-based biofuel cell biosensors. The introduction of nanostructured materials, such as gold nanoparticles (AuNPs) and metal–organic frameworks (MOFs), has dramatically increased electron transfer efficiency and optimized enzyme immobilization [36].

AuNPs, for example, provide excellent conductivity and a large surface area for enzyme loading, enhancing DET efficiency. MOFs, with their highly porous structures, support both electron transfer and substrate diffusion, making them beneficial for IET-based systems [37]. These nanomaterials enable biosensors to perform well even in complex biological environments, such as in vivo glucose monitoring or neurotransmitter detection in bodily fluids like sweat [38]. Furthermore, hybrid materials that combine conductive polymers, metal nanoparticles, and carbon-based nanostructures create synergies that enhance both electron transfer and enzyme stability. In addition, researchers are exploring bio-inspired strategies to optimize the enzyme-electrode interface. Functionalizing nanomaterials with biomimetic layers that replicate natural biological environments has been shown to enhance both DET and IET, as it improves enzyme orientation and maintains enzyme activity over extended periods [39].

### 2.3. Enzyme Immobilization Strategies

Over recent years, significant focus within the development of enzyme-based biofuel cells has centered on two primary concerns: the immobilization of enzymes and their stability when situated at the electrode interface. In the early stages of development, enzymes were utilized in solution within EBFCs. Later, there was a transition to placing them directly onto the electrode surface to enhance stability and efficiency. Initially, enzyme immobilization relied on adsorption, involving the incubation of enzymes in a solution. However, this method lacks stability as the enzymes tend to leach out during application in EBFCs. Recognizing this issue, Katz and Willner stressed the importance of covalent binding strategies to anchor enzymes onto gold electrode surfaces [40]. Concurrently, Karperien’s team experimented with enzyme immobilization via crosslinking within redox hydrogels [41]. This approach entailed the crosslinking of enzymes into polymer hydrogels, resulting in improved enzyme loading and increased stability compared to previous enzyme solutions [42]. However, it is essential to note that covalent binding or crosslinking procedures can potentially limit enzyme activity due to reduced freedom of movement.

**Figure 2:**
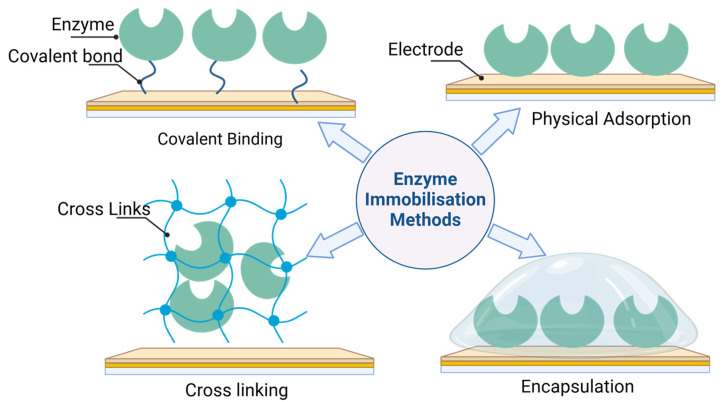
Methods for enzyme immobilization encompass physical adsorption, covalent attachment, cross-linking, and encapsulation.

This depiction highlights the need for optimization in the number of interconnections within redox polymer composite-based electrodes to advance BFCs effectively. Encapsulation and sandwiching techniques have also been employed for enzyme immobilization within a polymer layer on electrode surfaces [43]. These methods have been integrated over the past decade to craft more stable bioelectrodes. However, current challenges primarily revolve around the instability of cofactors or mediators, shifting the focus from enzyme stability [44]. The diverse array of techniques employed for enzyme immobilization is detailed in Figure 2.

#### 2.3.1. Physical Adsorption

Enzyme immobilization via physical adsorption involves the non-covalent binding of enzymes onto a substrate surface or matrix, relying on weak interactions such as van der Waals forces, hydrogen bonding, electrostatic forces, or hydrophobic interactions [45,46]. This method offers a relatively simple and gentle approach to attaching enzymes to different surfaces without altering their structure significantly [47]. The process begins by exposing the surface of a substrate, such as a solid support or a porous material, to an enzyme solution. The enzymes adhere to the surface through reversible interactions, forming a layer that can catalyze reactions while remaining physically bound to the substrate. This technique retains the enzyme’s native structure and functionality, as it does not involve chemical modification or covalent bonding. However, despite its simplicity, enzyme immobilization via physical adsorption has limitations. The main challenge lies in the stability and durability of the immobilized enzymes. Desorption from the surface, susceptibility to environmental conditions, and limited operational lifespan are common issues associated with this method. These challenges can affect the long-term efficiency and reusability of the immobilized enzymes. Researchers have explored various strategies to improve the stability of enzymes immobilized through physical adsorption. Substrate surface modifications using self-assembled monolayers (SAMs), nanomaterials, or specific molecules like thiols, surfactants, polyelectrolytes, or ionic liquids have been employed [48,49,50]. These modifications aim to create a more favorable environment, preventing denaturation, enhancing stability, and prolonging the activity of the immobilized enzymes. This framework created an enzymatic biofuel cell based on glucose and oxygen using mesoporous metal oxide electrodes immobilized with GOx and bilirubin oxidase (BOx) by physical adsorption. The EBFC showed a minimal decrease in output voltage after 30 h of continuous operation [51].

#### 2.3.2. Covalent Binding

The covalent binding method is widely acknowledged for its effectiveness and durability in enzyme immobilization, particularly on electrode surfaces. This approach leverages the reactivity of carboxyl and amino groups in enzymes, creating favorable conditions for forming covalent bonds [52,53]. Typically, this process involves the creation of amide bonds between amino and carboxylic groups, facilitated by activating agents such as 1-ethyl-3-(3-dimethylaminopropyl) carbodiimide (EDC) and N-hydroxysuccinimide (NHS), which help activate carboxyl groups and promote the formation of strong, stable bonds [54]. Covalent bonds provide several advantages, including establishing strong interactions between the enzyme and the electrode surface, resulting in improved stability compared to alternative methods like physical adsorption. It is worth noting that while the enzymatic activity may decrease slightly upon immobilization, covalent binding typically preserves a significant portion of the initial activity of the enzyme [55]. Expanding upon this, various studies have underscored the versatility and applicability of covalent binding in diverse enzymatic systems [56]. This technique has been widely used in biosensor development, where strong and durable enzyme immobilization is critical. Glucose oxidase has been covalently immobilized on gold electrodes modified with dendritic nanostructures using self-assembled monolayers, achieving enhanced stability and sensitivity in glucose biosensors [57]. Likewise, lactate oxidase immobilized onto graphene oxide and carbon nanotube surfaces has shown excellent performance in lactate sensing applications, further highlighting the utility of this technique. In enzymatic biofuel cells, where prolonged stability and activity are paramount, enzymes like laccase and bilirubin oxidase have been successfully immobilized on carbon-based electrodes through covalent bonding, achieving improved power output and operational lifetimes [58]. Recent progress in covalent binding techniques has led to developing porous structures such as MOFs and covalent organic frameworks (COFs). These materials offer extensive surface areas, customizable functional characteristics, and precise control over enzyme orientation, significantly improving the efficiency and stability of immobilized enzymes [59]. The durability afforded by covalent binding extends the operational lifespan of enzyme-based systems, making them suitable for continuous or prolonged use in industrial and environmental applications. Moreover, advancements in this technique continue to emerge, allowing for finer control over the orientation and density of immobilized enzymes, which is particularly beneficial for systems requiring precise enzyme–substrate interactions. For instance, the application of heterobifunctional cross-linkers has enabled the immobilization of enzyme arrays on microfluidic chips for multiplexed sensing, showcasing improved control over enzyme distribution and activity [60].

#### 2.3.3. Encapsulation

The encapsulation or entrapment technique for enzyme immobilization involves confining enzymes within polymer matrices, inorganic frameworks, or hybrid materials, which are often integrated onto electrode surfaces. This approach minimizes enzyme leaching while preserving their structural integrity and enzymatic activity by preventing denaturation and conformational changes. Among the various methods, sol–gel entrapment is frequently employed due to its ability to enhance both enzyme stability and catalytic activity [61,62]. This method has demonstrated considerable success in biofuel cell applications. For instance, a glucose/O_2_ EBFC employing a bioanode with carbon nanotubes (CNTs) for GOx immobilization and a biocathode with CNT-laccase showed a minimal 4% reduction in maximum power density after 30 days of storage in a buffered solution, highlighting the technique’s efficiency and durability [63].

Encapsulation within natural or synthetic membranes, such as agarose [64], lipids [65], and DNA [66], has also been extensively explored. These materials mimic the natural microenvironment of enzymes, ensuring proper folding and functional activity. However, their inherent low electrical conductivity often limits their applicability in bioelectrochemical systems. To overcome this limitation, conductive materials such as CNTs, 3-aminopropyltriethoxysilane (APTES), and ferricyanide are commonly co-encapsulated to facilitate efficient charge transfer, thereby improving the electrochemical performance of the system. Recent advancements have introduced composite bioelectrodes, such as those fabricated using multiwalled carbon nanotubes (MWCNTs), carbon fibers, and glucose dehydrogenase (GDH). These bioelectrodes exhibited remarkable stability, retaining 90% of their initial current response even after 90 days, demonstrating their suitability for long-term bioelectrochemical applications [67].

Additionally, the integration of CNT-loaded MOFs has emerged as a cutting-edge approach for enzyme encapsulation. MOFs provide a highly structured and porous crystalline framework that enhances enzyme stability, reusability, and activity. The exceptional properties of MOFs, including their high porosity, tunable chemical functionalities, and large surface area, make them ideal candidates for enzyme immobilization. These frameworks can accommodate enzymes in a controlled manner, influencing their orientation and catalytic efficiency while protecting them from harsh external conditions. This protective capability prevents enzyme denaturation, ensuring prolonged activity and functional stability. Furthermore, the controlled diffusion properties of MOFs address common challenges related to mass transfer limitations, enabling more efficient enzymatic reactions. Recent studies have demonstrated the ability of MOFs to encapsulate enzymes within their intricate networks, showcasing significant improvements in reusability and operational stability. By tailoring the pore sizes and surface properties of MOFs, researchers have achieved high enzyme loading capacities and optimized catalytic performance. For instance, encapsulation within MOF-based matrices has shown enhanced electron transfer and stability, making them indispensable in the development of advanced biofuel cells and biosensors [68,69,70]. To further advance enzyme encapsulation techniques, hybrid materials that combine the advantages of MOFs with conductive polymers or nanoparticles are being developed. These materials provide synergistic benefits, such as improved electron transfer rates, enhanced stability, and greater adaptability for multi-functional applications.

#### 2.3.4. Cross-Linking

Cross-linking is a widely employed and highly effective strategy for immobilizing enzymes onto electrode surfaces. This technique significantly enhances the stability and functionality of enzyme-based systems. It relies on bifunctional or multifunctional ligands to create robust enzyme networks, reducing enzyme leaching and ensuring improved long-term stability [71,72]. Covalent bonds formed between enzymes and cross-linking agents enhance the immobilized system’s mechanical strength and improve the durability of enzyme-based bioelectrodes, making them ideal for applications requiring extended operational reliability [73]. One prominent example of cross-linking is the use of Genipin, a natural and non-toxic cross-linker, as demonstrated by Hong et al. [74], to produce nano-cross-linked enzyme aggregates (nano-CLEAs) from Trametes versicolor laccase. Their research systematically analyzed the effects of various parameters, including cosolvent type, Genipin concentration, cross-linking duration, preparation pH, and amino group donor concentration, on the activity recovery of CLEAs.

Similarly, glutaraldehyde, one of the most commonly used cross-linking agents, has been extensively applied in preparing enzyme-based electrodes. It facilitates strong covalent linkages that enhance the thermal and chemical stability of the system. In a study by Qian et al. [75], Candida antarctica lipase B was first adsorbed onto silica nanoparticles and then cross-linked with glutaraldehyde, resulting in a cross-linked immobilized lipase with a recovery activity of 87.82%. Despite these advantages, cross-linkers can sometimes hinder electron transfer due to the random orientation of enzymes on the surface, which may slow down reaction kinetics. As previously discussed, enzyme immobilization plays a pivotal role in developing functional enzymatic electrodes, with critical factors including substrate choice, support material, and environmental conditions (e.g., whether the enzyme is immobilized on a matrix or within a hydrogel). These factors collectively affect the efficiency of the immobilization process, which in turn impacts the overall performance of enzymatic biofuel cells. When designing enzymatic biofuel cells, selecting the appropriate enzyme immobilization strategy is essential, as it directly influences the system’s stability and performance.

Each immobilization method presents unique advantages and limitations, affecting enzyme activity, stability, and electron transfer efficiency. Table 1 outlines various enzyme immobilization strategies to provide a comprehensive comparison, highlighting their respective benefits and drawbacks.

## 3. Nanomaterials for EBFCs

In the past fifteen years, there has been a notable upsurge in utilizing nanomaterials and nanoarchitectures, which have become indispensable in advancing bioelectrode design. Various porous structures derived from carbon-based materials such as carbon nanotubes, graphene, and carbon dot, along with noble metal, polymeric materials, and metal–organic frameworks, are employed as electrode materials and matrices for enzyme immobilization to enhance the performance of EBFCs. The exceptional characteristics of these porous nanostructures, including their large specific surface areas, high porosity, inherent conductivity, and biocompatibility, facilitate rapid electron and mass transport pathways crucial for efficient bioelectrocatalysis. This synergy of nanomaterials and bioelectrodes has opened up promising avenues in energy conversion and storage technologies, catering to diverse applications ranging from biomedical devices to environmental remediation and beyond [76].

### 3.1. Carbon-Based Nanomaterials

Carbon-based nanomaterials research has exploded in recent decades, opening up possibilities for electrode performance improvement in modern enzyme-based biosensors and biofuel cells. In this respect, a wide range of carbon-based nanomaterials CNTs, carbon nanodots (CNDs), bucky paper (BP), and 3D graphene have rapidly developed, indicating their versatility [77,78]. Therefore, these materials are highly desired due to their biocompatible nature, high conductivity, and exceptional mechano-thermal stability [79,80]. Under their diverse features that allow them to act as mediators of electron transfer and electrical wiring for enzymes facilitated by their extensive specific surface area, they have taken over bioelectrochemistry and related disciplines [81,82].

Carbon nanotubes, with their exceptional electrical, mechanical, and chemical properties, offer unique advantages in enhancing the performance of enzyme biofuel cells [83]. These cylindrical structures composed of carbon atoms provide a high surface area, excellent electrical conductivity, and biocompatibility, making them ideal candidates for enzyme immobilization and electron transfer mediation [84,85]. By integrating enzymes with CNTs, researchers aim to overcome the limitations of traditional enzyme biofuel cells and unlock their full potential for renewable energy generation. One of the key challenges in enzyme biofuel cell design is the stable immobilization of enzymes onto electrode surfaces. CNTs address this challenge by providing a three-dimensional scaffold for enzyme attachment, ensuring high enzyme loading and enhanced stability [86]. Moreover, the π–π stacking interactions between CNTs and aromatic amino acid residues in enzymes facilitate efficient electron transfer, thereby improving the catalytic activity and longevity of the enzyme biofuel cells. Another advantage is their ability to facilitate rapid electron transfer between enzymes and electrodes. The delocalized π-electron system of CNTs enables efficient charge transport, minimizing electron transfer resistance and enhancing the overall performance of enzyme biofuel cells. Additionally, functionalization techniques can be employed to tailor the surface properties of CNTs, promoting specific enzyme interactions and optimizing catalytic activity [87]. Until now, most high-performance bioelectrodes have been crafted using CNTs [88,89,90]. Additionally, CNTs serve as catalyst supports in EBFCs. CNTs were also utilized by Wang et al.000000 [91] as a matrix for the assembly of dehydrogenase toward the sensing of environmental contaminants. It exhibited an outstanding detection limit of 7.5 nM and peak power production of 15.3 μW/cm^2^. In another work, Yan et al. [92] reported an EBFC operating on glucose/O_2_ based on single-walled carbon nanotubes (SWNTs) to provide high output voltage. SWNTs play essential roles in this system, acting as materials of the electrodes and supports for the electrocatalysts and biocatalysts; among other, SWNTs allow direct electron transfer from laccase and enhance the biofuel cell’s efficiency.

CNTs, graphene oxide (GO), and its reduced (rGO) form have been identified as the most prospective nanomaterials for improving enzyme-catalyzed self-powered biosensors [93]. These materials are unique in their properties and can serve as excellent carriers for enzyme immobilization to further improve biosensing technologies. Kabir and co-authors, through their research works [94], have developed a new method of self-powered biosensing by using host electrodes based on two-dimensional mesoporous thermally reduced graphene oxide. For the bioelectrodes with immobilized enzymes, they used rGO-glucose oxidase at the bioanode and rGO-lactate oxidase at the biocathode. The rGO material was mesoporous, with a specific surface area of 179 m^2^ g^−1^ and a C/O ratio of 80:1. GOx native enzymatic activity was preserved in rGO from spectroscopic and electrochemical analyses. The rGO nanocomposites functionalized with GOx and LOx exhibited improved electrochemical properties when analyzed using cyclic voltammetry, with an estimated electron transfer rate constant of 2.14 s^−1^. The enzymatic biofuel cell achieved a maximum power density of 4.0 nW cm^−2^ and demonstrated stability over 15 days, maintaining a power output of approximately 9.0 nW cm^−2^ under ambient conditions.

**Figure 3:**
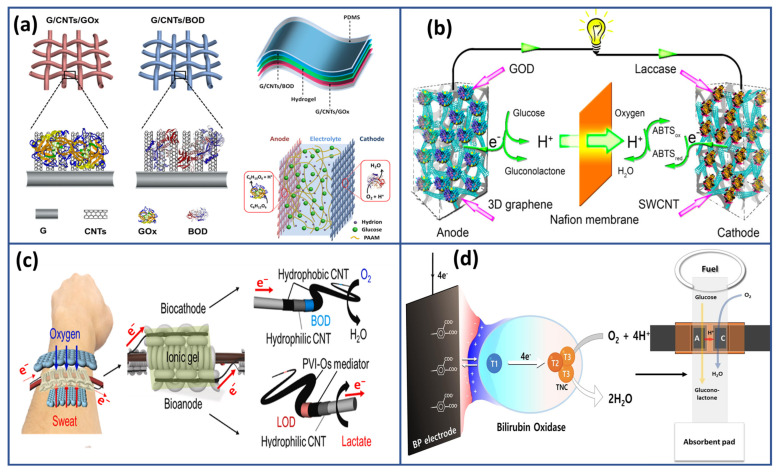
Applications of carbon nanomaterials in EBFC: (**a**) CNTs as a flexible substrate for GOx immobilization. Reproduced with permission from [95], (**b**) Combination of 3D graphene and CNT for GOD and laccase encapsulation. Reproduced with permission from [96], (**c**) hydrophobic CNT fiber as bioelectrode for BOx and laccase immobilization. Reproduced with permission from [97], (**d**) BP as bioelectrode for BOD electrografting. Reproduced with permission from [98].

In a similar vein, Chen and colleagues [95] pioneered a novel method to fabricate highly flexible and stretchable biofuel cells, holding significant potential for a broad spectrum of applications in flexible and wearable bioelectronics. Their approach involves the utilization of textile electrodes composed of a composite material consisting of graphene and carbon nanotubes (GO/CNTs), along with a polymer hydrogel electrolyte, ensuring excellent biocompatibility (Figure 3a). The carbon nanotube array, seamlessly integrated into a graphene layer, served a dual role, providing a conductive base for enzyme immobilization and establishing efficient pathways for charge transport between the enzyme and the graphene electrode. This innovative architecture resulted in exceptional performance, achieving an open-circuit voltage of 0.65 V and a power density of 64.2 μW cm^−2^, significantly exceeding previous benchmarks. Additionally, the unique design of the textile electrode, combined with the use of a polymer hydrogel electrolyte, enhanced the resilience of the biofuel cells, maintaining high power output even after 400 bending cycles and withstanding strains up to 60%. Furthermore, Prasad et al. [96] introduced a new EBFC configuration that utilized enzyme-functionalized 3D graphene along with single-walled carbon nanotube hybrid electrodes, employing glucose as the fuel and oxygen as the oxidant (Figure 3b).

These EBFCs demonstrated excellent stability, approaching the theoretical maximum open-circuit voltage of ~1.2 V, and achieved the highest recorded power density of 2.27 mW cm^−2^. Later, a groundbreaking biofuel cell bracelet was developed, capitalizing on the lactate present in human sweat [97]. This pioneering device features flexible fibers composed of enzymes, mediators, and CNTs. The biofuel cell consists of two main sections: a lactate oxidation segment utilizing an osmium-based mediator/CNT fiber and an oxygen reduction segment employing a bilirubin oxidase (BOD)/CNT fiber (Figure 3c). Notably, this layered cell demonstrated remarkable performance, generating 74 μW of power at 0.39 V when tested in a 20 mM artificial sweat lactate solution. Impressively, this level of performance is maintained at over 80% for 12 h. Furthermore, a booster six-cell bracelet variant has been developed to generate sufficient power at 2.0 V to operate digital wristwatches. This advancement holds significant promise for wearable energy harvesting technology, leveraging the abundant energy source provided by human sweat to power portable electronic devices.

Recently, Kim and his team [98] proposed an advanced biocathode design to enhance the performance of EBFCs based on using bucky paper as bioelectrode. Their approach incorporates BOD, 4-amino phthalic acid (4-APA), and a BP electrode, as shown in Figure 3d. By employing an electrografting technique, they augmented the electron transfer rate of BOD, forming a BP/4-APA structure with negatively charged surfaces. This configuration promoted a strong interaction between the positively charged T1 active site of BOx and the negative charges of BP/4-APA, thereby improving cathodic reaction reactivity and EBFC performance.

Evaluation of membrane-less paper-type EBFCs featuring the flexible BP/4-APA/BOx biocathode demonstrated impressive metrics, including a maximum power density of 92.025 μWcm^−2^ and an open circuit voltage of 61 mV under conditions mimicking living body fluid. These results underscore the crucial role of buckypaper in enhancing the efficiency and viability of biofuel cell technologies for potential applications in powering implantable devices within the human body.

CNDs are another type of carbon material known for their ability to promote enzyme immobilization on electrodes and facilitate bioelectrocatalysis, resulting in the generation of high-power outputs [99]. Extensive literature has highlighted the effectiveness of CNDs as platforms for immobilizing GOx on bioanodes and BOx on biocathodes. Experimental findings demonstrated that CNDs act as efficient electron mediators, thereby improving electron transfer kinetics. The fabrication process employed the drop-casting technique with Nafion as a binding agent. This ensured uniform enzyme immobilization on glassy carbon electrodes modified with carbon nanodots. The resulting EBFCs achieved a maximum power output of 40.8 μW cm^−2^ at 0.41 V. The open-circuit potential was recorded at 0.93 V. CNDs proved effective in enhancing enzyme immobilization and accelerating electron transfer. This makes them a strong candidate for improving EBFCs, biosensors, and hybrid self-powered systems [100].

Recent advancements EBFCs have utilized various hybrid nanomaterials to improve power density and catalytic efficiency. Glucose-based systems remain at the forefront of research, with notable examples including bioanodes based on chitosan with carbon nanoflakes, achieving a power density of 55 μW cm^−2^ [101], and Nafion-coated carbon nanofibers with graphene oxide composites, which reached 1.12 mW cm^−2^ [102]. Further innovations using graphene oxide combined with carbon nitride reported power densities of 0.57 mW cm^−2^ [103]. Systems employing gold nanoparticles and nitrogen-doped carbon nanotubes demonstrated enhanced performance, achieving up to 235 μW cm^−2^ [104].

Additionally, Fe_3_O_4_-functionalized composites achieved an impressive 1.32 mW cm^−2^ in glucose biofuel systems [105]. Also, bioanodes based on multi-MWCNTs and nanoflowers further demonstrated power densities as high as 200 μW cm^−2^ [106]. Ethanol-based EBFCs have also shown promise as an alternative energy source. For example, graphene-supported enzymatic systems achieved power densities of 5.16 mW cm^−2^ [107], while single-walled carbon nanotubes wrapped with reduced graphene oxide demonstrated substantial current densities of 165 μA cm^−2^ [108]. These findings highlight the potential of both glucose and ethanol as biofuels for energy harvesting in biosensors, each offering distinct advantages and challenges depending on the application.

Incorporating various carbon nanomaterials into EBFCs marks a notable advancement in bioelectrocatalysis. Referring to Table 2, a comprehensive compilation of recent studies on EBFCs utilizing carbon nanomaterial composites, it becomes apparent that carbon nanomaterials are crucial in optimizing the functionality of EBFCs. The data in the table highlights the impressive progress achieved in power density, open-circuit potential, and operational stability of EBFCs through the integration of carbon nanomaterials.

### 3.2. Noble Metals

Noble metals are highly regarded for their exceptional catalytic abilities and unique electronic configurations, making them essential in a wide range of applications, including biosensors [109], catalysis [110], and advanced electronics [111].

**Figure 4:**
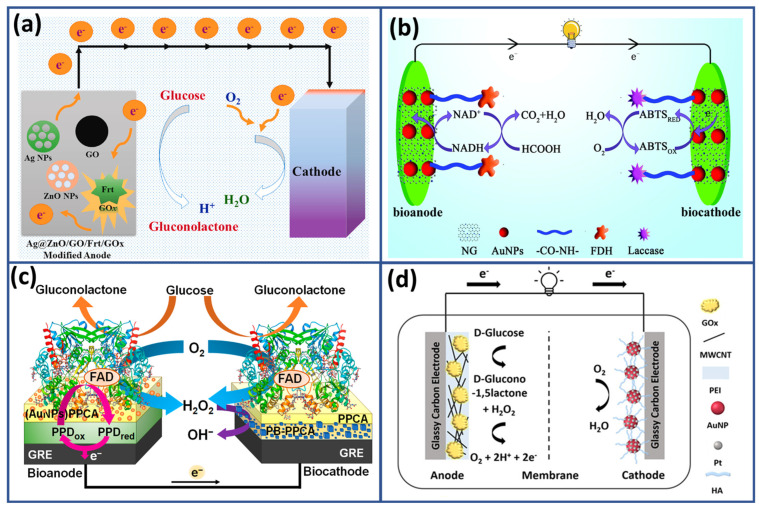
Applications of noble metals in enzymatic biofuel cell: (**a**) AgNPs anchored ZnO based bioanode operation. Reproduced with permission from [114], (**b**) AuNPs-embedded nitrogen-doped graphene bioanode for formate dehydrogenase integration. Reproduced with permission from [115], (**c**) AuNPs entrapped PPCA shell used as bioanode. Reproduced with permission from [116]. (**d**) AuNPs@PtNPs-based cathode electrode supports oxygen reduction. Reproduced with permission from [117].

Their natural talent for promoting catalytic reactions while maintaining stability is crucial in energy conversion. The importance of noble metals in EBFCs is highlighted by their ability to catalyze electrooxidation and electroreduction reactions that are fundamental to bioelectrocatalysis [112,113]. By providing active sites for these reactions, noble metals accelerate the rate of reactions, resulting in improved overall efficiency. Additionally, their inherent compatibility with biological systems ensures seamless integration, guaranteeing the stability and durability of EBFC devices in living environments. In a recent study, Ahamed et al. [114] presented a novel method for synthesizing a composite scaffold material. This material combines silver nanoparticles (AgNPs) with zinc oxide (ZnO) and is specifically designed for use in EBFCs, as depicted in Figure 4a. The proposed approach ensures the production of environmentally friendly materials and enhances their catalytic activity and stability. The composite material, Ag@ZnO-NP/GO, effectively facilitates electron transfer during bioelectrocatalysis. It achieved impressive current densities of 7.7 mA cm^−2^ at a glucose concentration of 30 mM. Another significant study by Gai et al. [115] introduced a nitrogen-doped graphene/gold nanoparticles/formate dehydrogenase (NG/AuNPs/FDH) bioanode (Figure 4b). This bioanode demonstrated efficient recycling of NAD^+^/NADH (nicotinamide adenine dinucleotide with hydrogen) during formic acid oxidation, resulting in a remarkable reduction in NADH oxidation overpotential. This advancement paved the way for the development of a membrane-less formic acid/O_2_ enzymatic biofuel cell that exhibits exceptional performance metrics. These included a maximum power density of 1.96 mW cm^−2^ and an open circuit potential of 0.95 V, with 80% maintenance over a 20-day period. Moreover, when two units were connected in series, the EBFC successfully powered red LEDs, demonstrating its practical applicability.

Furthermore, the research conducted by Minkstimiene et al. [116] reported the role of AuNPs in achieving an amperometric detection mechanism for glucose in biosensors powered by enzymatic biofuel cells. Their design incorporated glucose oxidase as the common enzyme for both the bioanode and biocathode. AuNPs were embedded within a poly(pyrrole-2-carboxylic acid) (PPCA) layer (Figure 4c). The anode was constructed using a poly(1,10-phenanthroline-5,6-dione) (PPD) nanocomposite, intricately combined with AuNPs and surrounded by a PPCA shell. The presence of AuNPs proved essential in enhancing the performance of the biosensor, particularly by improving electron transfer dynamics. This improvement resulted in increased sensitivity and selectivity in glucose detection. Additionally, the presence of AuNPs contributed to the durability and consistency of the biosensor, ensuring accurate results over a prolonged operational lifespan.

Furthermore, Han et al. [117] developed a bimetallic electrocatalyst composed of Au and Pt nanoparticles (AuNPs@PtNPs) to enhance the efficiency of the oxygen reduction reaction in EBFCs. This design presented in Figure 4d takes advantage of platinum’s modified electronic properties to improve oxygen reduction performance. Additionally, hyaluronate was used as a binder and stabilizer, enhancing the dispersibility, stability, and biocompatibility of the Au@Pt nanoparticles. These properties make the electrocatalyst especially suitable for wearable and implantable EBFC applications.

The optimized nanocomposite achieves an open circuit voltage of 0.35 V and a peak current density of 166 μA/cm^2^ at 0.04 V, highlighting its potential for effective energy generation in bioelectronic devices.

Noble metal catalysts have shown considerable promise in enzymatic biofuel cells, as evidenced by numerous studies highlighting their effectiveness in facilitating efficient electron transfer reactions and enhancing overall cell performance. However, their integration with enzymatic biofuel cells is susceptible to various limitations that can hinder their performance and overall sustainability. One major challenge is catalyst degradation, which occurs over time due to exposure to harsh operating conditions, chemical reactions, or fouling by impurities in the fuel or electrolyte. This degradation not only reduces the efficiency of the biofuel cell but also shortens its lifespan. Furthermore, noble metal catalysts can be easily poisoned by certain compounds present in the fuel or electrolyte. This poisoning leads to a significant decrease in catalytic activity, resulting in reduced power output and degraded overall performance of the biofuel cell. Moreover, these catalysts exhibit limited electrochemical stability, particularly under high temperatures, extreme pH levels, or high current densities. Such conditions can induce structural changes or corrosion in the noble metal catalysts, further compromising their performance and durability within the biofuel cell. In addition to technical challenges, environmental concerns surrounding the extraction and processing of noble metals pose significant issues. These processes often result in habitat destruction, water pollution, and high energy consumption, raising questions about the sustainability of their use in enzymatic biofuel cells. Addressing these limitations is crucial for the advancement and widespread adoption of enzymatic biofuel cell technology.

### 3.3. Conducting Polymers Based Bioelectrodes

Conducting polymers (CPs) are characterized by their unique feature of delocalized π-electrons within their polymeric chain backbone, which grants them exceptional electrical conductivity and a low ionization potential. These properties make CPs and their composites excellent electrode materials that ensure the efficient transfer of electron flow between enzymes and electrode surfaces in biosensor devices [118]. This conductive matrix enhances enzyme catalytic activity, leading to improved biosensor sensitivity and response times. Furthermore, CPs offer biocompatibility, ensuring minimal interference with enzyme function and biological systems. This allows for stable enzyme immobilization within the polymer matrix, ensuring long-term stability and reproducibility of biosensor measurements [119]. CPs, including polyindole, polythiophene, polyaniline, polypyrrole, and polyacetylene, are widely employed as supports for immobilizing enzymes to create high-performance electrodes.

**Figure 5:**
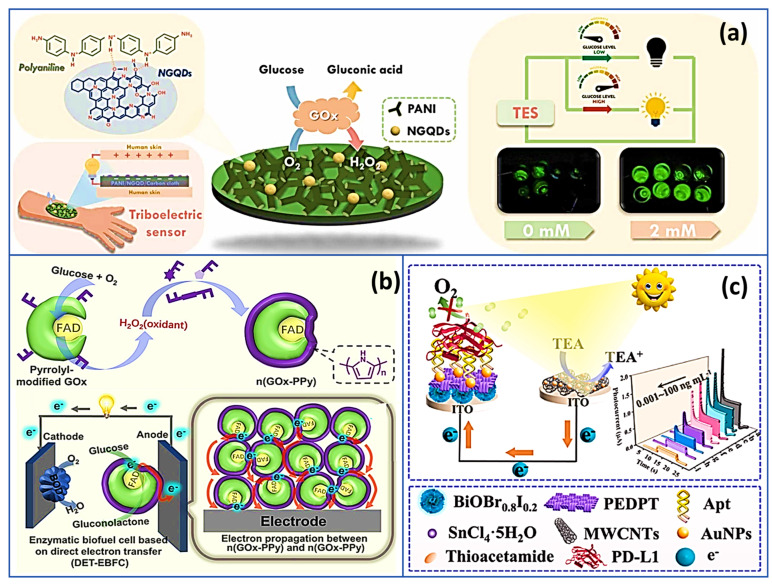
Applications of CPs in enzymatic biofuel cell: (**a**) Polyaniline as a flexible substrate for immobilization. Reproduced with permission from [121], (**b**) In situ growth of PPy for GOx encapsulation. Reproduced with permission from [122], (**c**) PEDOT integration in hybrid material for self-powered photoelectrochemical/visual sensing. Reproduced with permission from [123].

As one of the most commonly used conducting polymers, polypyrrole stands out for its remarkable ability to facilitate electron transfer, particularly with redox enzymes. This feature makes it a promising candidate for numerous applications. By incorporating cationic groups such as tris(bipyridyl)ruthenium(II), polypyrrole’s effectiveness in electrode performance is significantly boosted, with the complex serving as an electron relay [120]. Recently, Chang et al. [121] utilized polyaniline as a flexible substrate, serving as a matrix for immobilizing the GOx enzyme. The versatility of conducting polymers was further demonstrated by incorporating nitrogen-doped graphene quantum dots (NGQDs) into the nanocomposite structure, decorating the PANI and enhancing its functionality. To improve surface properties, electron-rich functional groups were introduced, increasing the surface electronegativity of PANI (Figure 5a). This modification facilitated more efficient charge transfer, which significantly boosted the triboelectric outputs of the system. The NGQDs provided additional pathways for electron transfer and increased surface charge, resulting in enhanced detection sensitivity. In comparison to a pristine PANI-GOx-based biosensor, the NGQDs-PANI-GOx nanocomposite exhibited superior performance in self-powered glucose detection, with a sensitivity of 23.52 μA mM^−1^, compared to 16.44 μA mM^−1^ for the unmodified system.

Subsequently, Huang and coworkers [122] focused on improving the conductivity and stability of EBFCs based glucose SPB. By triggering a self-encapsulation process, they were able to induce the formation of a polypyrrole (PPy) network within and around glucose oxidase upon glucose introduction, as shown in Figure 5b. This innovative method resulted in the creation of protective PPy shells encasing each GOx enzyme, significantly enhancing their stability. Moreover, the establishment of conductive connections between the active sites of GOx and the PPy network enabled a direct pathway for electron transfer. This increased the enzymatic activity and improved the efficiency of electron transport. As a direct outcome of employing the novel n(GOx-PPy) configuration, the EBFCs demonstrated a remarkable increase in power output, showing a 245-fold enhancement compared to traditional EBFCs.

In a recent study by Wang et al. [123], introduced an innovative dual-mode analytical device designed for the ultrasensitive detection of programmed death ligand-1 (PD-L1). The core of this sensor is an advanced organic-inorganic hybrid material, composed of poly(3,4-ethylenedioxythiophene) (PEDOT) combined with BiOBr_0.8_I_0.2_, which forms the cathode (Figure 5c). This hybrid material was meticulously engineered to optimize electron transfer processes, enhancing the detection efficiency without requiring additional energy inputs. The incorporation of PEDOT into the hybrid structure is particularly noteworthy for its role in increasing the electrical conductivity and structural stability of the cathode. This enhancement directly contributes to the overall performance of the sensing platform, allowing for more reliable and consistent detection.

Additionally, the functionalization of the cathode surface with a PD-L1-specific aptamer ensures a high degree of selectivity in targeting the PD-L1 protein, which is crucial for precise detection in biological samples. This sophisticated design, combining high conductivity, stability, and selectivity, enabled the device to achieve exceptional sensitivity. The detection limit for PD-L1 was remarkably low, reaching 0.29 pg/mL, even when tested in complex environments. In a further advancement, Prussian blue (PB) was integrated with MWCNTs/SnS_2_ to create a portable sensor chip for PD-L1 detection. Upon light exposure, photogenerated electrons from MWCNTs/SnS_2_ transfer to PB, causing a color shift from blue to white that visually indicates PD-L1 concentration. This self-powered detection system offers high sensitivity and ease of use, showcasing its potential as a practical tool for clinical diagnostics without the need for external power.

Another research team synthesized a nanocomposite of polythiophene-cobalt titanium oxide (PTH-TiO_2_) using oxidative chemical polymerization [124]. This PTH-TiO_2_ composite was a robust matrix for immobilizing the ferritin mediator and GOx, resulting in a durable and stable bioelectrode. The incorporation of ferritin played a key role in facilitating effective electron transfer between GOx and the conductive support material, significantly enhancing the bioanode’s performance. Electrochemical analyses, including linear sweep voltammetry and cyclic voltammetry, revealed a marked increase in the bioelectrocatalytic activity of GOx for glucose oxidation to gluconolactone. Notably, the PTH-TiO_2_/FRT/GOx bioelectrode achieved a current density of 7.8 mA cm^−2^ at a scan rate of 100 mV/s, highlighting its excellent catalytic efficiency. Conductive polymers offer numerous advantages for EBFCs, combining electrical conductivity, biocompatibility, and tunability. However, challenges such as maintaining stability in physiological environments and complex fabrication processes remain significant barriers to broader application. Incorporating metal–organic frameworks may provide a promising approach to enhancing the stability, tunability, and overall performance of these systems, potentially driving forward innovations in biosensing technologies and bioelectronics, particularly for health diagnostics.

### 3.4. Metal–Organic Frameworks Based Bioelectrodes

Metal–organic frameworks (MOF), also referred to as porous coordination polymers, represent a class of meticulously structured microporous crystalline materials formed from specific metal ions or clusters paired with organic linkers [125,126].

The inherent properties of MOFs, including their expansive surface area, high porosity, adjustable pore size, stability, straightforward synthesis methods, and catalytic activity, render them highly promising for a wide array of applications [127]. In recent years, MOFs have emerged as promising matrices for enzyme immobilization, owing to their unique attributes including tunable ultrahigh porosity, substantial specific surface areas and pore volumes, and considerable chemical, thermal, and mechanical resilience in specific environments [128,129,130]. The precisely ordered topologies, spanning from nanometers to micrometers, along with the uniform microenvironments within MOFs, render them adaptable and efficient hosts for various enzymes differing in dimensions, morphologies, surface functional groups, and charge distributions [131]. The integration of mesoporous MOFs with enzymes enhances stability in harsh conditions, as the ordered frameworks shield the enzymes, improving catalytic performance in challenging environments such as high temperatures, organic solvent presence, and extreme pH conditions [132,133]. Recent studies have demonstrated the potential of MOFs as ideal platforms for biomolecule immobilization in EBFC applications, showcasing improvements such as increased power density, prolonged operational life, and enhanced tolerance to environmental variables [134].

**Figure 6:**
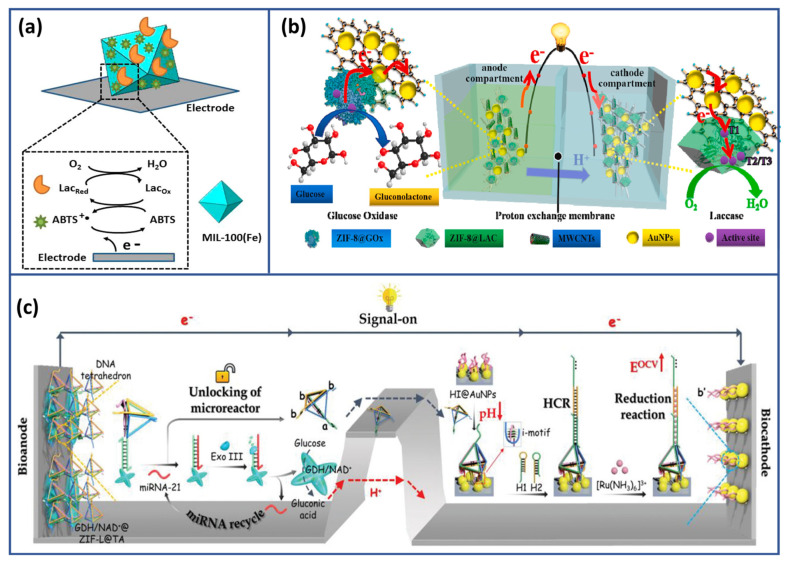
Applications of MOFs in enzymatic biofuel cell: (**a**) MIL-100(FeMOF) as immobilization matrix of laccase. Reproduced with permission from [135], (**b**) ZIF-8 as a host matrix for GOx. Reproduced with permission from [136], (**c**) ZIF-L as encapsulation matrix for GDH. Reproduced with permission from [137].

Patra and coworkers were the first to utilize mesoporous FeMOF, MIL-100, as an efficient immobilization matrix for laccase, aiming to construct biocathodes for oxygen reduction reactions (Figure 6a) [135]. In this system, the FeMOF matrix created an ideal environment for laccase immobilization, enhancing electron transfer dynamics and ionic transport, which resulted in a consistent and reliable electrochemical response. Laccase embedded within the MOF structure enabled efficient oxygen reduction using ABTS as a mediator, ensuring effective electron transfer at the electrode interface. This FeMOF-based biocathode system demonstrated robust enzyme stability and long-term functionality. Later, another research group [136] investigated the direct synthesis of ZIF-8 on electrospun cellulose acetate nanofiber membranes, embedding GOx. Additionally, MWCNTs together with AuNPs were sequentially deposited onto the ZIF-8@enzyme membranes to produce flexible conductive electrodes. This system was incorporated into an autonomous biosensor setup for glucose detection, as illustrated in Figure 6b.

The proposed glucose sensors demonstrated notable enhancements in durability, maintaining uninterrupted operation for as long as 15 h. The direct synthesis of MOFs on cellulose nanofibers, combined with the incorporation of enzymes into MOFs, presented an innovative strategy for creating robust and dependable EBFC-based biosensors.

Similarly, Yan et al. [137] utilized the large porosity of ZIF-L to design an encapsulation framework for GDH, aiming to construct a self-sustaining biosensor for miRNA-21 detection (Figure 6c). This system used a microreactor mechanism triggered by the presence of miRNA-21, which unlocked the DNA tetrahedron structure. The bioanode facilitated glucose oxidation, while the biocathode completed the reduction reaction, generating a detectable signal. The biosensor exhibited remarkable sensitivity with a detection limit of 2 aM, successfully identifying cancer cells and analyzing serum from breast cancer patients. These developments showcase the versatile and efficient nature of MOF-based platforms in creating advanced biosensors for various applications, including glucose monitoring and cancer detection. While MOFs demonstrated high performance as platforms for enzyme immobilization, it is important to acknowledge certain limitations that may affect their widespread applicability. One significant drawback is the stability issues associated with MOFs. Although they may exhibit excellent stability in certain conditions, MOFs can degrade activity over time in harsh environments, affecting long-term performance [138]. Additionally, the cost and complexity of synthesizing MOFs challenge their widespread adoption. The labor-intensive and expensive nature of MOF synthesis, along with the complexity of modification processes, may hinder scalability. Furthermore, the pore sizes and structures of MOFs may not always be optimal for efficient mass transfer of substrates and products, potentially reducing the catalytic efficiency of immobilized enzymes and limiting biosensor performance. Moreover, MOFs may have limited capacity for enzyme loading, restricting the amount of enzyme that can be immobilized and impacting biosensor sensitivity and detection limits.

## 4. EBFCs Based Wearable SPB for Sweat Analysis

The integration of biosensing technologies with EBFCs represents a groundbreaking advancement in the realm of bioelectronics. This synergy has paved the way for a new era characterized by real-time monitoring and detection of a diverse array of biomarkers essential to both physiological and pathological processes [139]. Among these biomarkers acetylcholine, lactate, glucose, and ethanol stand out as focal points in the evolution of SPBs [140]. This innovation harnesses the inherent energy derived from biological substrates, enabling these biosensors to operate autonomously. The exploration of EBFC-based biosensors capabilities in detecting and quantifying these crucial biomolecules not only signifies a monumental leap forward in sensor technology but also holds immense promise for transforming healthcare, diagnostics, and biomedical research [141]. By leveraging the energy naturally harvested from biological sources, these biosensors offer a sustainable and self-sufficient approach while opening up new avenues for advancements in precision medicine and personalized healthcare. This convergence of biosensing and biofuel cell technologies has the potential to revolutionize the landscape of medical monitoring and research, ushering in a future where diagnostics are highly accurate, environmentally conscious, and self-sustaining [142].

### 4.1. Self-Powered Neurotransmitter Biosensor

Acetylcholine (ACh) is one of the earliest neurotransmitters to be identified, found in both the peripheral and central nervous systems [143]. It primarily facilitates the transmission of signals from motor neurons to muscles, particularly in key organs such as the heart, bladder, and stomach. Additionally, ACh plays an essential role in functions such as memory, cognition, and movement. Consequently, precise measurement of ACh levels is critical for diagnosing and treating certain medical conditions [144]. The typical concentration of acetylcholine in whole blood is around 1.26 ng mL^−1^ (approximately 4.623 nM) [145]. In 2017, researchers from the United Kingdom and Portugal introduced the first membrane-less SPB capable of detecting acetylcholine in real time and in situ [146]. This biofuel cell employed an enzymatic anode coupled with a platinum cathode, creating a hybrid system. The researchers created an acetylcholinesterase-immobilized electrode using gold with a high porosity as the electrode material. The process of immobilization was both effective and simple, eliminating the need for harsh chemicals or complex pretreatment methods. The enzyme-functionalized electrode was integrated into a compact, membrane-free fuel cell, which exhibited strong sensitivity to varying acetylcholine concentrations. This fuel cell achieved a peak output of 4 nW at 260 mV and demonstrated a current density of 9 µA cm^−2^.

Additionally, it had a detection threshold of 10 µM and an average response time of 3 min, highlighting its potential for real-time monitoring and point-of-care diagnostic applications. These promising findings have significant implications for point-of-care Alzheimer’s diagnosis, offering a potential alternative to labor-intensive sample treatments and costly instruments. The uniqueness of this biosensor lies in its successful detection of ACh and its self-powered design. While there has been significant progress in biosensors for other analytes, such as lactate and glucose, the exploration of SPBs for neurotransmitters is still in its infancy. This work represented an important step towards understanding and harnessing the potential of self-powered neurotransmitter biosensors. However, the limited focus on SPBs for neurotransmitters poses challenges in technological development and understanding the intricacies of neurotransmitter dynamics.

Achieving high selectivity and sensitivity, especially in the complex matrix of biological samples, remains a significant hurdle. Additionally, ensuring the stability and reliability of such biosensors over extended periods, especially in vivo, is a challenge that necessitates further exploration. As the only known work in the field, the ACh biosensor catalyzes future research endeavors. The current emphasis on ACh opens the door for broader investigations into SPBs for other neurotransmitters. Given that neurotransmitters can be found in bodily fluids such as sweat, expanding the scope of research to include these accessible matrices presents exciting possibilities for non-invasive monitoring. Looking ahead, the future trajectory of research in this domain could involve enhancing the efficiency of Acetylcholine biosensors and addressing the existing challenges. Researchers may explore innovative solutions, including novel electrode materials, advanced immobilization techniques, and miniaturization for improved integration with biological systems. The transition from ACh to other neurotransmitters, particularly those present in sweat, could provide new avenues for developing wearable and non-invasive biosensors for real-time monitoring. Overcoming existing challenges and expanding the repertoire of neurotransmitters under investigation could lead to transformative developments in biosensing technology, offering new possibilities for diagnostics and personalized medicine.

### 4.2. Self-Powered Glucose Biosensor

**Figure 7:**
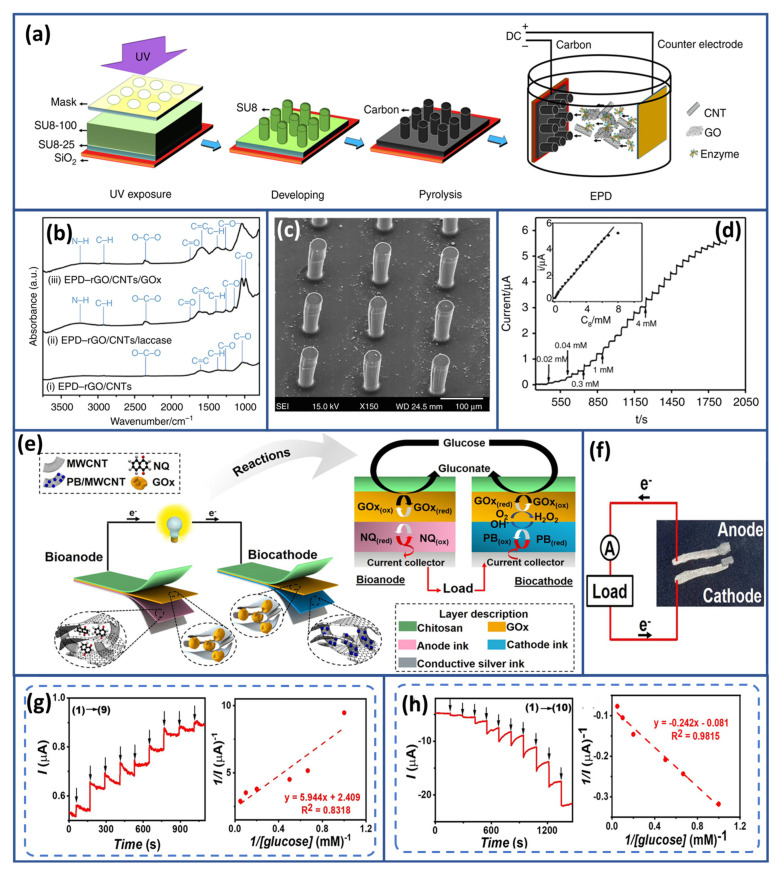
Glucose SPB-Based EBFCs: (**a**) illustration of the assembly process for the 3D micropillar array platform, (**b**) FTIR spectra illustrating the immobilization stages, (**c**) SEM images depicting the morphology of the rGO/CNT/GOx-coated 3D carbon micropillar array, (**d**) amperometric response of the rGO/CNTs/GOx bioanode, with the inset showing the response to successive glucose. Reproduced with permission from [149], (**e**) key components of a screen-printed glucose BFC, emphasizing the vital redox reactions at both the bioanode and biocathode, (**f**) schematic diagram of the screen-printed biosensing electronic system, (**g**,**h**) amperometric response and calibration curves showing the current response for both the GOx/NQ/MWCNT-based bioanode and biocathode, respectively. Reproduced with permission from [153].

Glucose, a primary energy source, profoundly influences human health. Blood glucose levels exceeding 1000 mg L^−1^ (around 5.56 mM) after fasting often indicate potential diabetes, necessitating further testing like the 2 h glucose tolerance test. Conversely, levels below 70 mg L^−1^ (3.89 mM approximately) signal hypoglycemia, leading to adverse effects if left untreated. Balanced glucose levels are crucial for sustained energy, brain function, and cellular activities. Hormones like insulin and glucagon tightly regulate blood glucose. Diabetes disrupts this balance, leading to chronic high glucose levels and severe health complications. Managing glucose levels through lifestyle, diet, exercise, and medical interventions is key to preventing and managing metabolic disorders, underscoring the critical role of glucose in overall well-being [147]. Given the fundamental role of glucose in health and its implications for diseases, extensive research has long been devoted to developing glucose biosensors. Current efforts are particularly directed towards advancing wearable sensors for self-powered functionalities. Enzyme-based biofuel cells are emerging as promising candidates in this pursuit. Their potential to harness enzymatic reactions to generate energy renders them an attractive option for powering wearable glucose sensors, paving the way for convenient and sustainable continuous monitoring of glucose levels. In 2016, Choi and coworkers introduced an economical self-powered glucose biosensor [148]. The proposed biosensor operated on a glucose/O_2_ biofuel cell platform, utilizing a chitosan/GOx-based bioanode for glucose oxidation and a Ni-activated carbon cathode for oxygen reduction. To efficiently incorporate the system onto paper, the researchers cleverly employed a three-dimensional origami-inspired design for constructing the BFC. The output power of these glucose biosensors showed a clear relationship with glucose concentration, spanning from 1 mM to 5 mM. Moreover, the biosensor exhibited notable sensitivity of 0.02 mA mM^−1^. This biosensor offered significant advantages: cost-effectiveness, independence from external power sources or complex transducers, and swift, reliable result generation. Such qualities positioned this biosensor as ideal for clinical diagnostics and environmental monitoring, especially in resource-constrained regions of the world. Following this, Song and Wang [149] made significant strides in developing closed-loop micro EBFC systems designed specifically for glucose SPBs. Their enabling platform deployed three-dimensional carbon micropillar arrays coated with a composite of rGO, CNTs, and a biocatalyst. A hybrid approach to advanced fabrication uses top-down carbon micro-electro-mechanical systems (C-MEMS) and bottom-up the approach of the electrophoretic deposition techniques employed in forming the bioelectrodes. Illustrated in Figure 7a is the initiation of the fabrication where 3D micropillar arrays were first fabricated employing a two-step photolithography process and then pyrolyzed. These 3D carbon micro-pillar arrays are further subjected to enzyme-coated nanocomposite depositions using the EPD technique.

The composite of enzyme and nanomaterial depositions on C-MEMS-based micropillar arrays is also demonstrated by Scanning Electron Microscopy (Figure 7b). Individual micropillars, having a height of approximately 120 µm and a diameter of about 35 µm, with center-to-center spacing of 130 µm, are shown. FTIR. The FTIR analysis is another convincing tool for portraying the immobilization of GOx and laccase on the EBFC’s anode and cathode. Figure 7(bi) represents FTIR spectra of rGO and CNTs on the micropillars where no peaks were noticed. In contrast, Figure 7(bii) represents after the deposition of rGO/CNTs/laccase on the biocathode and shows several absorption peaks at different wave numbers confirming the existence of the enzymes. Furthermore, the FTIR spectrum Figure 7(biii) of deposited rGO/CNTs/GOx on 3D carbon micropillar arrays at the bioanode offers strong characteristic peaks, which add weight to the successful immobilization of the enzymes in the rGO/CNT composite. Such a bioanode would be highly responsive to characteristic changes based on the glucose concentration, and its linearity would be obtained for its response to the glucose value range from 0.02 mM to 7.24 mM, as shown in Figure 7d. The theoretical performance of the rGO/CNT-integrated EBFC system was analyzed using finite element modeling, which proved highly effective in evaluating its efficiency and performance. The experimental findings were encouraging, showing that the system achieved a peak power density of 196.04 µW cm^−2^ at 0.61 V, nearly doubling the output compared to EBFC systems based solely on rGO. Furthermore, the observed power density reached 71.1% of the theoretical value, reflecting high system efficiency. This innovative approach, combining the strengths of rGO and CNTs, significantly enhanced the electrochemical performance of the EBFC, paving the way for more efficient glucose-based SPBs. The results indicate that further optimization of enzyme immobilization techniques, along with the strategic use of nanocomposites, holds great potential for developing next-generation biofuel cells with high power density and efficiency.

Chansaenpak et al. presented a novel biofuel cell-based glucose sensing strategy [150]. They exploited redox enzymes such as GDH, GOx and horseradish peroxidase (HRP), anchoring them as biocatalysts on specially designed electrodes. To enhance biocatalyst immobilization, these electrodes were reinforced with additional polymers derived from MWCNTs and rGO. A key advantage of this design is its use of glucose as both anode and cathode substrate, which makes it more compact and durable; second, the system can operate under air-saturated conditions without requiring gas purging; and third, the system sensitivity is increased by integrating carbon nanostructures with multiple enzyme cascades. Such an approach enables precise glucose detection across a broad range, making it exceptionally suitable for analyzing common biofluids and industrial food samples. Additionally, Cho et al. [151] unveiled a pioneering self-powered glucose biosensor that integrates screen-printed biofuel cells into a Band-Aid-like adhesive patch, designed for non-invasive glucose tracking through human sweat. This biosensor consists of a graphene/chitosan/glucose oxidase bioanode for glucose oxidation and a nickel/activated carbon cathode for oxygen reduction. Notably, they incorporated a reservoir of conductive bioanodes treated with poly(3,4-ethylene-dioxythiophene): polystyrene sulfonate to construct an enzymatic glucose/oxygen counter-flow biofuel cell, vertically stacked within the Band-Aid dressing adhered directly to the skin. Through capillary action, the reservoir efficiently gathers sweat, enabling glucose concentration monitoring without reliance on external power sources or complex reading instruments. This glucose biosensor boasts a broad linear range, extending from 0.02 to 1.0 mg mL^−1^ (approximately 0.11 to 5.56 mM). It offers a sensitivity of 1.35 µA mM^−1^, marking a notable 70-fold improvement over previously mentioned self-powered counterparts.

In the same framework, Ding et al. [152] recently introduced a fingertip-wearable microgrid system for autonomous glucose detection in sweat, integrating enzymatic biofuel cells and AgCl-Zn batteries to harvest and store bioenergy. This self-powered device continuously monitors metabolites without external energy sources. Utilizing osmosis and a paper fluidic system, it maintains a steady sweat supply to the sensor array for reliable, on-demand detection. The compact design incorporates biofuel cells, batteries, a flexible circuit board, and wearable sensors, enabling seamless metabolic monitoring. Low-power electronics handle signal acquisition and wireless data transmission, making it a promising tool for diabetes management.

Furthermore, Slaughter and co-workers [154] successfully engineered a glucose biosensor that operates on self-generated power. They achieved this by combining a charge pump and capacitors with BFCs. In this approach, bioanodes consist primarily of pyrroloquinoline quinone (PQQ) and GDH, while biocathodes are powered by laccase. Under ideal circumstances, the biosensor achieved a maximum power density of 67.86 μW cm^−2^. Additionally, it demonstrated the capability to detect glucose within a linear range of 0.5 to 45 mM. Building upon this achievement, the same research group proposed a subsequent self-powered biosensing platform for glucose detection [155]. This advanced platform featured a PQQ-GDH-modified CNTs bioanode and BOD-modified CNTs biocathode, along with the integration of a capacitor circuit and charge pump with the BFC. This improved biosensing system exhibited a linear dynamic range for glucose detection from 0.5 to 70 mM. Notably, the second biosensing platform showcased substantial improvements.

The sensitivity of the charge/discharge frequency increment in response to glucose concentration reached 37.66 Hz mM^−1^ cm^−2^, which was up 2-fold higher than their initial work. This enhancement in sensitivity reflected the research team dedication to refining the biosensor performance, offering a more accurate and efficient means of glucose monitoring. The integration of advanced components and the expanded linear dynamic range underscore the continuous progress in self-powered glucose biosensor technology, paving the way for improved applications in glucose detection and monitoring.

Recently, Veenuttranon et al. [153] introduced a screen-printed glucose biofuel cell (BFC) for self-powered glucose detection, utilizing nanocomposite inks. The bioanode consists of GOx, NQ, and MWCNTs, while the biocathode includes PB and MWCNTs. The BFC operates through redox reactions at both the bioanode and biocathode, with glucose oxidation occurring at the anode and H_2_O_2_ reduction at the cathode, generating electrical power from glucose metabolism. The key components are depicted in Figure 7e, illustrating the redox reactions at the bioanode and biocathode. Figure 7f provides a schematic diagram of the screen-printed biosensing electronic system, showing the connection between the bioanode and biocathode for energy harvesting. The amperometric response of the bioanode was evaluated using various glucose concentrations, as shown in Figure 7g. The current response increased with rising glucose levels, confirming efficient glucose oxidation by the GOx/NQ/MWCNT-based bioanode. The calibration curve showed a linear response between 1.5 and 20 mM, with a sensitivity of 0.0073 μA mM^−1^. Similarly, Figure 7h displays the amperometric response of the biocathode, which demonstrated increased current with higher glucose concentrations, attributed to the PB electrocatalyst facilitating H_2_O_2_ reduction. The calibration plot was linear in the 1.5 to 20 mM range, with a sensitivity of approximately 0.4738 μA mM^−1^. With its effective energy harvesting and glucose-sensing capabilities, this screen-printed glucose BFC presents a promising solution for self-powered biosensors, suitable for integration into wearable devices for continuous glucose monitoring.

### 4.3. Self-Powered Lactate Biosensor

Lactate, a vital component of our body’s energy dynamics, is generated when the muscle tissue breaks down internal glycogen using the glycolytic pathway, leading to the formation of pyruvate, subsequently converted by lactate dehydrogenase [156]. This production outpaces consumption, causing a proportional increase in lactate concentration correlating with the level of physical exertion [157]. Notably, during periods of heightened physical activity, the rate of lactate production surpasses its utilization, leading to a proportional increase in lactate concentration, an indicator closely tied to the level of exertion. Beyond its role as an energy source, lactate significantly influences the signaling of the body’s metabolic status. Lactate traverses the body via specific transporters, becoming detectable in bodily fluids like saliva, urine, serum, tears, and sweat. In these fluids, lactate concentrations vary, approximating levels of 0.1 mM, 0.5 mM, 1 mM, 3 mM, and 20 mM, respectively. This variability in concentrations offers valuable insights into the body’s metabolic responses during different activities and exertion levels [158].

**Figure 8:**
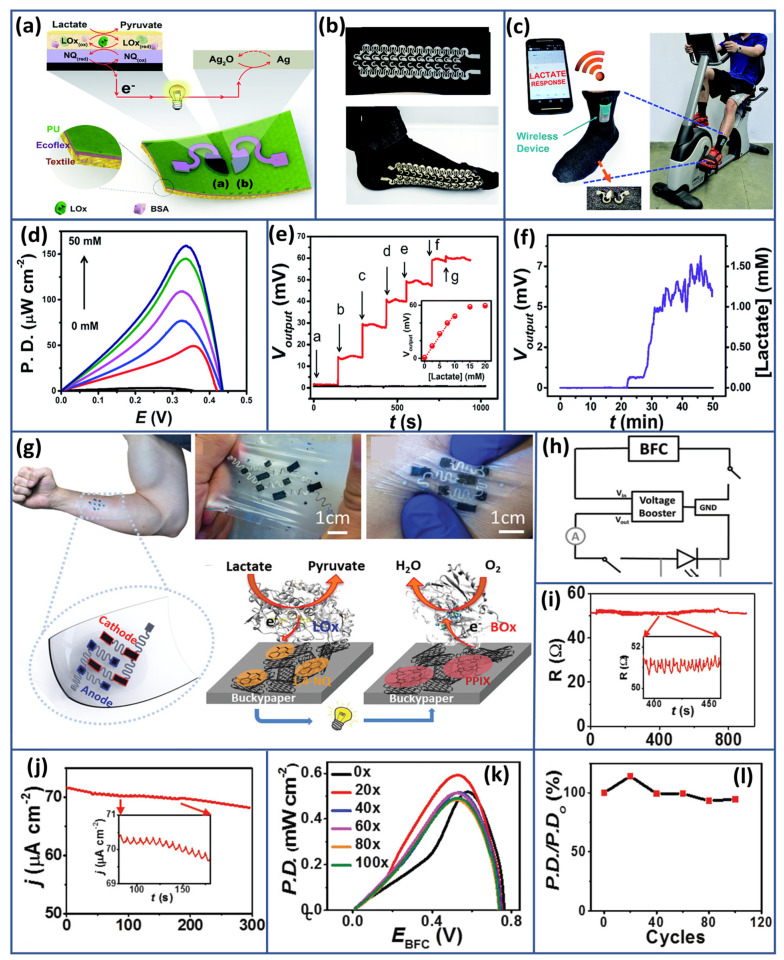
EBFCs based lactate SPB: (**a**) Diagram illustrating the flexible lactate biofuel cell and electrochemical reactions at the anode and cathode, (**b**) image of the flexible biofuel cell array printed on fabric for use as SPB in wearable socks, (**c**) photo of a volunteer wearing the sock-based biofuel cell array during exercise, (**d**) graph depicting power density versus potential for the stretchable glucose biofuel cell at various glucose concentration, (**e**) voltage output generated by the lactate SPB and a compact wireless device, (**f**) real-time monitoring of lactate levels during a cycling exercise test on the body. Reproduced with permission from [160], (**g**) Adaptable BFC device on a human arm with diagrams illustrating energy production via sweat lactate oxidation and O_2_ reduction, (**h**) circuit schematic for using the flexible epidermal BFC patch to power an LED through a DC-DC converter, (**i**) resistance profile during a 20% stretch, with inset displaying resistance fluctuations, (**j**) current density output with inset showing current density fluctuations, (**k**) power density versus voltage graphs for various cycle counts in lactate, (**l**) percentage change in power density at 0.55 V across multiple stretching cycles. Reproduced with permission from [164].

Progress in the dynamic realm of modern sports medicine has sparked increasing interest in devising continuous and user-friendly techniques for lactate detection. Furthermore, research within the broader medical sphere has correlated lactate concentration with diverse biological processes and medical conditions, such as tumor cell metastases and head trauma [159]. In 2016, Wang and his team [160] made a significant advancement in the development of SPBs by creating biofuel cells integrated into textiles for wearable applications. Their innovative approach involved screen-printing mechanically robust inks onto a flexible substrate of Ecoflex^®^ and polyurethane (PU), each 75 μm thick, applied in a serpentine pattern for enhanced flexibility. This serpentine design allows the biofuel cell to endure mechanical deformation, such as stretching, twisting, and compression, making it ideal for wearable technology. To fabricate the biofuel cell, the researchers applied Ag/AgCl and CNT-based ink, forming the CNTs-Ag/AgCl-PU-Ecoflex^®^-textile substrate. The cathode, composed of stretchable Ag_2_O/Ag, was created by electrooxidizing the Ag/AgCl, CNTs, and Ecoflex^®^ mixture. The bioanode was produced by electrooxidizing the substrate and then applying a drop-cast of 1,4-naphthoquinone (NQ), CNTs, and lactate oxidase, as illustrated in Figure 8a. This setup enabled printing multiple biofuel cells on the substrate, generating a biofuel cell array designed for self-powered lactate sensing.

Figure 8b shows the biofuel cell array integrated into a wearable sock, demonstrating the potential of this technology for real-world applications. The flexibility of the design ensures the device remains operational during movement. Figure 8c shows a volunteer wearing the biofuel cell sock during physical activity. The sock-based biofuel cell system is connected to a wireless device, highlighting its ability to transmit data in real-time. The performance of the glucose biofuel cell was evaluated by varying glucose concentrations, as depicted in Figure 8d, which demonstrates the power density (P.D.) curve for different glucose concentrations. The power density increases proportionally with glucose levels, with a maximum power density of 160 μW cm^−2^ at 0.3 V and an open-circuit voltage of 0.44 V. The response of the system to lactate concentration changes is shown in Figure 8e, where real-time voltage output (V_output) is displayed for different lactate concentrations over time. The device shows a clear increase in voltage with rising lactate concentrations, as confirmed by the inset, which reveals a linear relationship between lactate concentration and voltage output. This demonstrates the biofuel cell’s capability for continuous lactate monitoring in real-time conditions.

Moreover, Figure 8f shows the voltage output response to lactate concentrations during continuous cycling. As the volunteer’s lactate levels increased with prolonged exercise, the biofuel cell responded by generating a corresponding voltage output, effectively illustrating its ability to monitor metabolic activity in real-time. One of the most innovative aspects of this work is the use of nanomaterial-based inks and serpentine structural designs, which impart high flexibility and durability to the bioelectronic devices. These design features enable the biofuel cells to maintain their functionality under various mechanical deformations, such as stretching, twisting, and compression, making them well-suited for wearable applications. The combination of nanomaterials and flexible designs ensures the resilience and robustness of the biofuel cells during real-life movements, such as exercise, without compromising performance. In terms of performance, both the glucose and lactate biofuel cells, using a single enzyme and membrane-free configuration, achieved notable power outputs. Glucose biofuel cell reached a power density of 160 μW cm^−2^ with open-circuit voltage 0.44 V, whereas in lactate biofuel cell the power density was 250 μW cm^−2^ with open-circuit voltage 0.46 V. Remarkably, even after 100 cycles of 100% stretching, the biofuel cells maintained both their structural integrity and stable power output. These stretchable, self-powered textile sensors are capable of generating power signals proportional to the substrate concentration, making them highly effective for integration into wearable devices for real-time biosensing and energy harvesting applications.

In the same year, Hickey et al. [161] presented a novel method of immobilizing LOx on a carbon electrode with dimethylferrocene-modified linear polyethylenimine (FcMe_2_-LPEI) and launched a highly sensitive lactate biosensor. This substantially increased the current density and allowed them to work as an integrated biocathode, which was using the Box enzyme that had been established. The biofuel cell thus produces adequate electrochemical energy for lactate detection without having to depend on external power sources. The sensor also shows highly sensitive features registering a sensitivity value of 45 µA cm^−2^mM^−1^ over the entire range of lactate concentration from 0 mM to 5 mM. Furthermore, FcMe_2_-LPEI/LOx tasted remarkably high characteristic performances that were power density, 122 µW cm^−2^; current density, 657 µA cm^−2^ and open circuit potential, 0.57 V. This establishes the biofuel cell as an effective tool for lactate detection while also serving as a reliable power source for low-scale electronic devices. Following that, Baingane and Slaughter presented a self-powered electrochemical lactate biosensor that operated lactate/O_2_ biofuel cells [162]. The biosensor design showcased a biocathode incorporating BOx-modified CNTs for efficient oxygen reduction, paired with a bioanode containing LDH-modified CNTs for effective lactate oxidation. A 10pF capacitor was incorporated into the BFC, connected through a charge pump circuit, to support real-time lactate monitoring. The energy produced by the BFC was accumulated in the capacitor via the charge pump circuit until a predetermined discharge start voltage was reached. Afterward, the capacitor released its charge until the voltage decreased to the discharge stop threshold, initiating a recharge cycle. Interestingly, the rate of charging and discharging was closely tied to lactate concentrations, within the range of 1 to 100 mM. However, after two weeks, the BFCs experienced a 56.5% decrease in power output from their initial levels, with an overall reduction of 76.9%. Specifically, the electrical output of 1 mM lactate decreased by 52.2% at the end of week one and by 86.8% at the end of the second week. These results highlight the importance of recalibrating or replacing the system every two weeks to maintain consistent accuracy and efficiency.

Another research group introduced the first self-powered, fully screen-printed skin patch lactate biosensor with a visual readout, eliminating the need for external devices [163]. This innovative biosensor features a lactate oxidase-based anode and an osmium-polymer cathode, both printed on a transparent PEDOT polystyrene-sulfonate electrode. To ensure separation between the cathode and the lactate-sensing anode, an ion-gel layer composed of poly(vinylidene fluoride-co-hexafluoropropylene), a gelling agent, and an ionic liquid (EMIM-Tf) was incorporated. This layer acts as an effective ion barrier, preventing unwanted interference while optimizing ion transport.

This design provides real-time, non-invasive lactate monitoring with immediate visual feedback via an electrochromic display. The transparent PEDOT electrode provides a clear output and serves as an effective sweat lactate indicator. By modifying the enzyme system of the anode, the device can detect different analytes. The PEDOT cathode maintains over 90% transparency without affecting the PB color change. The display shows lactate levels from 0 to 10 mM with a contrast ratio of 1.43. Although the total response time is up to 24 min, 85% of the color change occurs within the first 10 min, providing fast and visible results.

In addition, Wang’s team has developed a pioneering wearable device that generates electrical energy from sweat [164]. This patented technology can continuously power an LED, significantly advancing self-sustaining, eco-friendly wearable electronics. Central to this innovation is a flexible lactate/O_2_ biofuel cell with BP electrodes made of multi-walled carbon nanotubes. The printed patch adheres seamlessly to sweat-prone areas of the skin, such as the arm, neck, chest, and back, maintaining excellent conformity (Figure 8g). The integration of the patch with a voltage booster, LED, and switch is shown in Figure 8h. The mechanical stability of the BP BFC was tested using a biaxial stretching stage controlled by a stepping motor. After 100 stretching cycles, the circuit’s resistance remained low, demonstrating the patch’s robustness (Figure 8i). Further tests monitored the BFC’s discharge current under constant load, showing minimal change over 20 stretching cycles (Figure 8j). The power output remained consistent after 100 cycles (Figure 8k,l). These electrodes were integrated into a stretchable screen-printed current collector with steady operation under mechanical deformation. The biofuel cell on the volunteer’s forearm exhibits a peak power of 450 µW, and the functionality is such that after incorporating a voltage booster system, this specifically illuminates an LED in both pulsed and continuous modes during physical activity, which is a typical demonstration of its efficacy as self-sustaining energy towards prospective wearable devices.

### 4.4. Self-Powered Ethanol Biosensor

**Figure 9:**
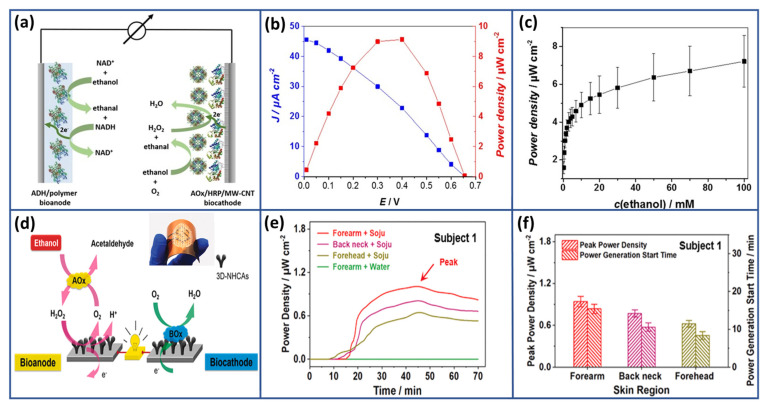
EBFCs based Ethanol SPB: (**a**) schematic illustrating the design of the bioanode and biocathode, along with the redox reactions at each electrode, (**b**) representative current density (blue) and power density (red) obtained for constant ethanol (0.5 M) and NAD^+^ (10 mM) concentrations, (**c**) calibration curves depict the power density at the BFC against ethanol concentration. Reproduced with permission from [89]. (**d**) Illustration of the 3D-NHCAs-based ethanol/oxygen BFC, (**e**) Real-time power density mapping of subject 1 across different skin regions during cycling, (**f**) correlation between peak power density and the initiation time of power generation for the epidermal ethanol BFC across various skin regions. Reproduced with permission from [168].

Ethanol, a key component of alcoholic beverages and widely used in various industries, poses health risks with both short-term effects like intoxication and dehydration and long-term consequences such as addiction and organ damage. Excessive consumption increases susceptibility to diseases like cirrhosis and cardiovascular conditions, impacting mental health with elevated risks of depression and anxiety. Ethanol also holds legal significance, notably in traffic incidents [165,166]. In the People’s Republic of China, a blood alcohol concentration of 2 g L^−1^ (∼43 mM) marks the threshold for considering individuals as driving under the influence (DUI) offenders, emphasizing the role of ethanol measurement in assessing impairment and ensuring public safety. Given the wide-ranging impact of ethanol on health and its legal implications, there is a pressing need for cutting-edge solutions. Developing a real-time, self-powered ethanol biosensor becomes paramount, offering continuous monitoring to detect potential impairments promptly [167]. This innovation holds the potential to significantly enhance public safety by addressing the evolving challenges associated with ethanol consumption. For this purpose, Schuhmann’s research team [89] devised a self-sustaining ethanol biosensor by employing a biofuel cell architecture. In such design, a bioanode modified with ADH and a biocathode modified with AOx and HRP were associated. Such a biocathode made it possible to carry out oxidation of ethanol to acetaldehyde in the presence of O_2_, which was reduced to H_2_O_2_, then further converted to H_2_O by the action of HRP, through releasing two electrode-supported electrons (Figure 9a).

Meanwhile, the toluidine blue (TB) mediator within the TB-PVP matrix facilitated the progress of NAD^+^, occurring under ADH’s action to transform ethanol into acetaldehyde and lower NAD^+^ into NADH, in the presence of TB as a mediator between TB-modified polyvinylpyrrolidone (PVP) matrices. The TB-PVP matrices not only entrapped the enzyme ADH but also shifted the potential for the TB mediator to some more positive value. The authors illustrated the sensitivity of the biosensor by highlighting the power density and current density features of the fully assembled SPB under constant ethanol concentration, as shown in Figure 9b. Additionally, calibration curves displaying power density against different ethanol concentrations up to 100 mM confirmed the reproducibility of the developed biosensor (Figure 9c).

The proposed biofuel cell exhibited an open-circuit voltage of approximately 0.66 V, and its sensing capabilities were further validated by analyzing ethanol content in commercial liquor using the standard addition method. This affirms its potential for on-site monitoring of real samples.

The high open circuit voltage makes this system particularly promising for developing new ethanol-powered energy conversion technologies. In a recent study, Sun et al. [168] introduced the first flexible and wearable microfluidic system that collects ethanol/oxygen from exogenous sources and operates in real-time on the human body. This innovative system generates electrical power from the perspiration of alcohol drinkers.It integrates a skin-interfaced microfluidic module for the continuous collection, transportation, storage, and excretion of fresh sweat. Additionally, it incorporates a flexible ethanol/oxygen BFC module, enabling non-invasive, real-time bioenergy generation (Figure 9d). The BFC module’s efficiency is derived from its enzymatic solid-phase extraction arrays, consisting of three key functional layers, including electrode material, anodic and cathodic enzymes, and a crosslinking agent. These layers ensure efficient biofuel electrooxidation, oxygen electroreduction, electron transfer acceleration, and long-term stability. Using high-flexible materials like PI and PET for the substrates ensures good adhesion and practical power generation on different skin regions. The research showed that a superior power output was achieved when the device was worn on the forearm, while the lowest was on the forehead. The study also demonstrated effective sweat bioenergy collection under various practical scenarios.

A volunteer, after consuming a bottle of Soju, wore the device on their forearm during a 70 min biking session. Power generation started after 15.23 min and reached its peak of 1.01 μW cm^−2^ at 45.23 min, compared to negligible power output when only water was consumed (Figure 9e). This confirmed that sweat could be successfully used to harvest power, emphasizing the importance of alcohol intake. Testing was conducted on various body parts, as shown in Figure 9f. In this study, time-lapsed power density measurements over 30 min revealed that the forehead began generating power after just 8.33 min, although it produced the lowest peak power density at 0.65 μW cm^−2^. In contrast, the forearm demonstrated the highest peak power density of approximately 1.01 μW cm^−2^. This noninvasive e-skin ethanol biofuel cell eliminates the risks of biotoxicity and tissue trauma typically associated with invasive BFCs. It also presents a novel approach for harvesting bioenergy from human sweat after alcohol consumption, overcoming the limitations of wearable BFCs reliant on endogenous fuels like glucose or lactate. This establishes a groundbreaking method for noninvasive, on-body electricity generation from sweat.

## 5. EBFCs-Based Implantable SPBs

EBFCs have transcended their role in wearable technology to emerge as promising power sources for implantable and microscale devices, despite their relatively modest power output. The utilization of EBFCs to fuel implantable SPBs holds significant potential across various biomedical domains. Integrating EBFCs into implantable SPBs offers numerous advantages. EBFCs efficiently harness energy from biological fluids like blood or interstitial fluid, prolonging device operation without requiring frequent battery replacements or external charging. This feature is particularly advantageous for medical implants, where battery replacement surgeries pose risks and inconveniences to patients. Moreover, EBFC-based SPBs provide a renewable and sustainable energy source by utilizing naturally occurring substrates such as glucose or lactate found in bodily fluids. This capacity ensures uninterrupted power generation, enhancing the longevity of implantable devices. In 2010, researchers from Université Joseph Fourier in Grenoble conducted a groundbreaking study that marked a significant advancement in implantable biosensors [169]. They were able to implant the first operating Glucose Biofuel Cell (GBFC) in the retroperitoneal space of freely moving rats. This was achieved by a new family of GBFCs that employ a novel mechanical confinement method for enzymes and redox mediators.

Compared to traditional methods, the key innovation of this approach lies in its ability to incorporate various enzymes and redox mediators, which led to a simpler GBFC design and significantly increased the quantity of active enzymes. The most efficient GBFC in the study utilized composite graphite disks for both the anode and cathode, incorporating glucose oxidase, ubiquinone, polyphenol oxidase (PPO), and quinone. Notably, PPO effectively catalyzed the reduction in dioxygen to water under physiological conditions, further enhancing the overall cells efficiency and suitability for bioelectrochemical applications. This specific GBFC achieved a power peak of 24.4 µW mL^−1^, surpassing the requirements of pacemakers and suggesting potential applications in the development of implantable artificial organs. The study also conducted a stability assessment of the GBFC over a 40-day period, demonstrating its long-term viability. The concept of mechanical confinement opens up possibilities for seamless integration of enzymes or redox mediators, paving the way for GBFCs to generate tens of mW or even higher power outputs.

**Figure 10:**
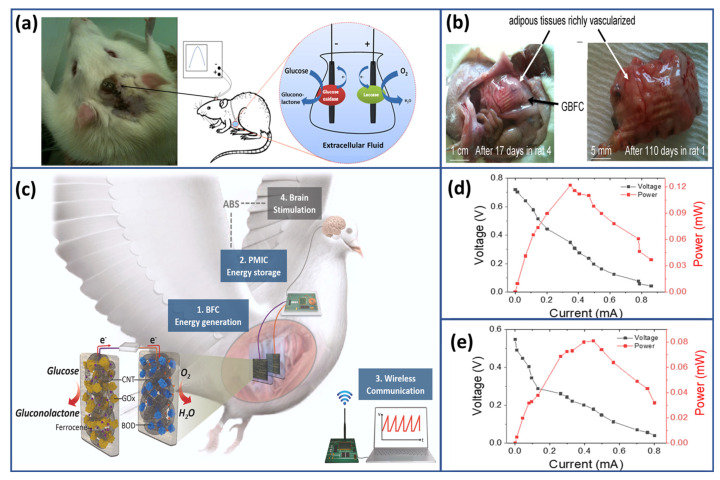
EBFCs-based implantable SPB: (**a**) schematic illustrating the electrical setup of the implanted GBFC in a Wistar rat coupled with enzyme reactions, (**b**) recovered GBFCs shown after 17 and 110 days of implantation in the rat brain. Reproduced with permission from [170], (**c**) Incorporation of BFC and ABS in a pigeon for wireless power management, (**d**) in vitro measurements, and (**e**) in vivo evaluations of BFC performance using a wireless power meter. Reproduced with permission from [171].

In 2013, Zebda et al. [170] achieved a significant milestone by demonstrating the first biofuel cell functioning in vivo within a mammalian system, which provided a continuous, self-sustained power supply for small electronic devices. This GBFC utilized enzyme-modified electrodes immobilized on carbon nanotubes, with GOx at the bioanode and laccase at the biocathode, facilitating oxygen reduction (Figure 10a). The biofuel cell was tested in vivo with an initial open-circuit voltage of 0.57 V after implantation into the abdominal cavity of a rat. A notable power output of 38.7 μW corresponding to a power density of 193.5 μW cm^−2^ and a volumetric power of 161 μW mL^−1^. These outputs were sufficient to power small devices like an LED and a digital thermometer, showcasing the practical viability of this technology for implantable applications. A most stable performance was noted of the implanted GBFC over an extended time. No signs of immune rejection or inflammation were seen in the rat over 110 days of implantation, indicating a quite high degree of biocompatibility (Figure 10b). This study was the first to show the practicability of enzymatic biofuel cells functioning in living organisms to power small electronic devices, which opens up prospective application in implantable medical technologies: biosensors, pacemakers, drug delivery systems. This work set the groundwork for further research on more advanced and long-lasting biofuel cells for continuous in vivo energy harvesting. Recently, Lee et al. [171] demonstrated the feasibility of their fully implantable Biofuel Cell-Animal Brain Stimulator (BFC-ABS) system in flying birds. Their innovative work addressed the challenging task of providing electrical power to microelectronic circuitry implanted within the bodies of small and lightweight avian species (Figure 10c). Recognizing the vulnerability of birds to device implantation, particularly due to the complex faradaic redox processes inherent in biofuel cells, the authors devised an effective solution. Their study showcased the successful integration of a BFC and an ABS within a pigeon model.

By harnessing enzymatic reactions of glucose oxidation and oxygen reduction, the BFC efficiently generated electrical power to drive the ABS. Notably, in vitro (Figure 10d) and vivo experiments (Figure 10e) yielded a power output of 0.08 mW, solely utilizing the natural glucose and oxygen resources within the body of pigeon, highlighting the practicality of the system. Furthermore, the authors overcame the theoretical constraints of BFC power output by incorporating external circuits to store and manage energy, ensuring a consistent power supply for neurostimulation. Through the use of advanced materials like MWCNT and aminoferrocene enclosed in biocompatible cellulose dialysis membranes, they achieved durable and biocompatible components for the BFC. This achievement represents a significant advancement in the realm of bioelectronic medicine, opening up new possibilities for self-powered neuromodulation in avian subjects. Moreover, it holds promise for extending similar applications to diverse species, potentially including humans, thus marking a notable milestone in the field. Menassol et al. [172] presented an innovative study introducing an iron/nitrogen co-doped reduced graphene oxide (Fe/N-rGO) cathode tailored for glucose biofuel cells.

Through a systematic fabrication process utilizing mechanical compression, these cathodes exhibit long-term stability, surpassing two years with a mere 8% loss in activity. Comprehensive cytotoxicity assessments under standard in vitro cell -culture conditions affirm the cathodes’ biocompatibility, offering promising prospects for in vivo applications. This crucial finding underscores the feasibility of employing Fe/N-rGO cathodes in biomedical settings. Furthermore, this study included in vivo experimentation, where these cathodes were successfully implanted for three months in a rat model. Remarkably, the implanted cathodes exhibited sustained performance and compatibility, reaffirming their potential for real-world physiological contexts. The incorporation of crosslinked chitosan as a binder enhances cathode stability, a critical aspect highlighted by both in vitro and in vivo evaluations. This dual approach, combining in vitro biocompatibility assessments with in vivo implantation studies, establishes a robust framework for future implantable biofuel cell technology development.

However, significant challenges hinder the scalability of EBFCs for practical applications [173,174]. One major issue is enzyme degradation due to the lack of cell membrane protection and direct exposure to the reaction medium. Additional obstacles include enzyme protection, electron transfer rate, and enzyme selection, all of which need optimization to enhance EBFC performance. At present, the use of EBFCs in vivo remains restricted, as they primarily serve as in vitro power sources within the milliwatt range [175]. This constraint is largely attributed to inefficient electrical interaction between the enzyme and the conductive substrate [176]. To address these issues, researchers are exploring several strategies. One approach involves developing protective coatings or membranes to prevent enzyme degradation and improve their stability in implantable self-powered therapy systems [177]. Efforts are also being made to enhance electron transfer kinetics and select the most appropriate enzymes.

Enhancing the electron transfer between the enzyme and the conductive substrate remains a key focus area for researchers seeking to advance EBFC technology. This involves the utilization of advanced electrode materials, nanostructured interfaces, and innovative enzyme immobilization techniques. These advancements aim to enhance electron transfer rates and overall power output of EBFCs for in vivo applications. In this context, Belgacem’s group [178] has developed high-performance 3D-printed cathodes for implantable biofuel cells. Their research focuses on optimizing ink formulation using nanocellulose-chitosan and N-doped graphene. These cathodes demonstrate a remarkable 3.6-fold enhancement in current density compared with traditional counterparts. The group successfully assembled biofuel cells with these cathodes and enzymatic anodes, achieving the utmost current and power and densities of 320 μA/cm^2^ and 80 μW/cm^2^, respectively. Furthermore, implantation in rats showed no severe inflammatory reactions, suggesting biocompatibility. To provide a clearer perspective on the potential of EBFCs for wearable and implantable applications, Table 3 presents a comparative analysis of biofuel energy sources, their advantages, limitations, and the challenges that need to be addressed. This table highlights the distinct requirements for wearable and implantable devices, as their energy budgets and operational conditions differ significantly.

Recently De la Paz et al. [179] developed a battery-free, self-powered wireless biosensing capsule for dynamic glucose monitoring in the small intestine. It utilizes a glucose biofuel cell (BFC) to generate energy and measure glucose levels based on power variations. Data transmission is achieved via energy-efficient magnetic human body communication (mHBC) in the 40–200 MHz range, minimizing energy dissipation. The capsule detects glucose within 3–90 mM (limit: 4.656 mM) and remains stable for three hours in artificial intestinal fluid. It also exhibits strong selectivity against common interfering substances, ensuring reliable glucose sensing. While glucose remains the most widely utilized energy source due to its abundance and stability in body fluids, other biofuels such as lactate, ethanol, and neurotransmitters offer niche advantages but also pose unique challenges.

## 6. Challenges and Trends

Over the past three decades, significant strides have been made in the development of biofuel cells. Despite these advancements, numerous challenges persist, hindering the widespread commercialization of enzymatic biofuel cells across diverse applications, as discussed in this review. Current research underscores that EBFCs exhibit suboptimal electrochemical efficiency, incomplete fuel oxidation, limited power output, and operational instability. These limitations collectively affect key performance parameters, including power density, current density, overall efficiency, energy density, and catalytic activity, often failing to meet the required performance thresholds [180]. While multiple solutions exist for each issue, implementing remedies can be challenging due to potential trade-offs, complicating efforts to enhance cell performance. Given the interconnected nature of these difficulties, a comprehensive and systematic analysis is necessary to pinpoint the root causes behind each problem, facilitating effective resolution strategies [181].

Scientists have directed significant efforts toward enhancing the stability and performance of EBFCs, often focusing on material engineering and biotechnological approaches to improve the enzyme environment at the electrode surface. However, stabilizing strategies that enhance material robustness can sometimes compromise enzyme activity, ultimately affecting the overall electrochemical performance. To tackle this, several strategies have been employed, such as loading higher quantities of enzymes onto the electrode surface, increasing enzyme catalytic activity, and accelerating electron transfer between enzymes and electrodes. These methods aim to boost power and current density, which are critical for more practical and efficient EBFC systems. A crucial challenge remains enzyme denaturation and degradation, particularly within the harsh physiological environments found in medical applications. As such, future research must explore advanced protein engineering techniques to develop enzymes with enhanced thermal and chemical stability. Alongside this, the incorporation of nanomaterials such as carbon nanotubes, graphene, and metal–organic frameworks offers protective environments that can improve enzyme stability and activity. These nanomaterials not only provide a conducive matrix for enzymes but also enhance the overall conductivity and electron transfer within EBFC systems.

While these advancements show promise, incomplete fuel oxidation remains a significant barrier to efficiency. Innovations in multi-enzyme cascades, which mimic natural metabolic pathways, are emerging as a solution to this problem by enabling more complete fuel oxidation [182,183]. In parallel, co-factor regeneration systems and enzyme co-immobilization approaches have demonstrated potential in maintaining high catalytic turnover rates, thus improving fuel utilization. Optimizing the spatial arrangement of these enzymes on electrode surfaces also holds great promise for enhancing fuel efficiency. Achieving higher power and current densities is a key goal in making EBFCs more practical for real-world applications. One approach is to increase enzyme loading on the electrodes by using 3D nanostructured materials that offer a larger surface area for enzyme attachment [184]. Additionally, enhancing enzyme catalytic activity through genetic modifications or artificial enzymes can further boost power output. Facilitating faster electron transfer between enzymes and electrodes, achieved through conductive nanomaterials and redox mediators, has been shown to significantly improve EBFC performance.

Despite these innovations, the scarcity of oxygen in biological fluids and buffers continues to be a major challenge for EBFC oxygen cathodes. This issue is compounded by the presence of peroxide, generated by the oxidase enzymes in the anode, which negatively affects cathode performance [185]. To address this, adopting air-breathing biocathodes, commonly used in conventional fuel cells, could be a promising solution. Furthermore, developing three-phase boundaries for bilirubin oxidase and laccase-based biocathodes remains an underexplored area that requires further investigation. Progress in this field could unlock commercial applications for portable biobatteries and implanted biofuel cells.

In addition to these material and catalytic innovations, a key consideration in EBFC development is the integration of external circuits or power management systems. These systems are essential for harvesting, storing, and regulating the variable power output of EBFCs. Smart power management circuits can dynamically adapt to fluctuations in EBFC-generated power, ensuring a stable supply of energy for biosensors and other medical devices. The ability to efficiently store and distribute energy is especially important in wearable or implantable devices, where continuous operation is critical. Furthermore, incorporating hybrid energy-harvesting systems, such as piezoelectric materials, can enhance the overall energy output. Piezoelectric components convert mechanical energy, like body movements, into electricity, supplementing the power generated by EBFCs. This hybrid approach is particularly valuable in environments where stable, long-term power is required, such as in wearable health monitoring systems [186].

To ensure the safety and biocompatibility of EBFCs in medical applications, the materials used must be non-toxic and designed to avoid adverse immune responses. Research should also focus on biocompatible electrode materials and effective encapsulation strategies to prevent harmful interactions with the body. Long-term in vivo studies are necessary to evaluate the chronic effects and overall safety of implanted EBFCs [187]. Furthermore, for EBFCs to achieve commercial viability, scalable and cost-effective manufacturing techniques need to be developed. Advances in microfabrication and nanotechnology will be instrumental in miniaturizing and integrating EBFC systems for broader use. For EBFCs to truly meet the specific energy requirements and operational conditions of diverse applications, a tailored approach is required. Optimization of enzymes, fuel sources, and electrode materials must be aligned with the intended use, whether for continuous glucose monitoring, neural stimulation, or other biomedical applications. Advancing EBFC technology will rely on effective cross-disciplinary collaboration among bioengineers, materials scientists, and medical professionals to design application-specific systems tailored to the unique needs of each scenario. Future research should also focus on integrating EBFCs with emerging technologies such as artificial intelligence (AI) and machine learning. These technologies could optimize energy management, predict enzyme degradation, and improve performance efficiency, paving the way for more robust and adaptive EBFC systems

Finally, cost considerations remain a critical challenge, as the production of bioelectrodes and specialized enzyme formulations is still expensive. Reducing the cost of materials while maintaining efficiency and biocompatibility will be essential for large-scale deployment. Additionally, scalability presents another major hurdle, as current fabrication techniques may not be suitable for mass production without compromising enzyme stability and electrode performance. Developing standardized manufacturing protocols and scalable fabrication methods will be crucial in bringing EBFCs from laboratory research to real-world applications. Market acceptance is another key factor influencing the future of EBFC commercialization. While EBFCs offer unique advantages such as self-sustainability and biocompatibility, they must compete with well-established power sources such as lithium-based batteries in biomedical applications. Demonstrating the long-term reliability, safety, and performance consistency of EBFCs in real-world conditions will be critical to gaining regulatory approvals and fostering industry adoption. Furthermore, collaboration between researchers, industry partners, and regulatory bodies will be necessary to streamline the transition from research to commercial product development [188].

## 7. Conclusions

EBFCs have demonstrated considerable potential as sustainable energy sources for wearable and implantable biosensors, advancing medical diagnostics and therapeutic technologies. This review highlights key progress in enzyme immobilization techniques, nanomaterial innovations, and enzyme–electrode interactions, all of which contribute to enhancing EBFC performance, stability, and integration in real-world applications. Despite these advancements, significant challenges remain in optimizing power output, extending operational lifetime, and ensuring reliable performance in physiological environments. A particularly promising direction for EBFC research lies in the continued development of advanced nanomaterials, such as graphene, carbon nanotubes, and metal–organic frameworks, which have improved electron transfer rates, stabilized enzymes, and enhanced power efficiency. These innovations have played a pivotal role in overcoming the traditional limitations of EBFCs, bringing them closer to practical applications in biosensing platforms. Looking ahead, the transition from experimental prototypes to commercially viable EBFC-based devices requires overcoming key challenges related to scalability, biocompatibility, and cost-effective manufacturing. Future research must focus on designing compact, reliable, and long-lasting EBFC systems capable of sustained operation in complex environments, such as the human body. Key areas for further development include improving enzyme durability, optimizing electrode surface properties, and integrating protective coatings that enhance stability without compromising catalytic efficiency. Additionally, interdisciplinary collaboration will be crucial in advancing EBFC integration with hybrid energy-harvesting systems and self-powered biosensors, paving the way for autonomous medical devices. The potential of EBFCs to revolutionize real-time health monitoring and therapeutic interventions is vast. With continued advancements in material science, biotechnology, and system integration, EBFCs could emerge as a cornerstone technology in next-generation wearable and implantable medical devices. Addressing the current limitations through focused research and innovation will be key to unlocking their full potential. Ultimately, bridging the gap between laboratory research and real-world applications will determine the success of EBFCs in shaping the future of bioelectronics and personalized healthcare.

## Figures and Tables

**Table 1:** Comparison of enzyme immobilization strategies: advantages and disadvantages.

Immobilization Strategies	Advantages	Drawbacks
**Physical Adsorption**	Simple and quick method; Minimal alteration to enzyme structure; Reversible process	Weak enzyme attachment; Potential desorption over time; Limited stability and reusability
**Covalent Binding**	Strong and stable enzyme attachment; Enhanced reusability; Greater resistance to environmental conditions	Chemical modification of enzyme may affect activity; Labor-intensive and time-consuming process; Limited enzyme loading capacity
**Encapsulation**	Protects enzyme from harsh environments; Allows for controlled release; Good stability and reusability	Diffusion limitations for substrates and products; Potential mass transfer issues; Complex fabrication processes and materials
**Cross-Linking**	Provides stability to enzymes; Allows for moderate reusability; Retention of enzyme activity	Limited control over cross-linking density; Possibility of enzyme inactivation during cross-linking; Can alter enzyme conformation and activity

**Table 2:** Performance of EBFCs based carbon nanomaterial hybrid composite.

Bioanode	Biocathode	Biofuel	Current Density	Power Density	Ref
**CS/GOx/CNCs/GCE**	Pt sheet	Glucose	434 μA cm^−2^	55 μW cm^−2^	[101]
**Nafion/GOx/PANI1600@GO/GCE**	CNFs/GCE	Glucose	2.48 mA cm^−2^	1.12 mW cm^−2^	[102]
**GOx/CNTs@C_3_N_4_/GCE**	LOx/N-CNTs@C_3_N_4_/GCE	Glucose	1.21 mA cm^−2^	0.57 mW cm^−2^	[103]
**rGO/AuNPs/N-doped CNTs**	Pt electrode	Glucose	NR	235 µW cm^−2^	[104]
**GCE/PPy/Au/CNT@Fe_3_O_4_/FRT/GOD**	Au Electrode	Glucose	6.01 mA cm^−2^	1.32 mW cm^−2^	[105]
**Graphene/SPE/GDH**	Graphene/SPE/LOx	Ethanol	88 μA cm^−2^	5.16 μW cm^−2^	[107]
**GCE/MWCNT/GOx nanoflowers**	GCE/MWCNT/LOx nanoflowers	Glucose	1.62 mA cm^−2^	200 μW cm^−2^	[106]
**SWCNTs wrapped with rGO/Alcohol dehydrogenase**	NR	Ethanol	165 μA cm^−2^	NR	[108]

Chitosan (CS), carbon nanochips (CNCs), glassy carbon electrode (GCE), platinum (Pt), ferritin (FRT), not reported (NR).

**Table 3:** Comparison of biofuel energy sources for wearable and implantable biosensors.

Energy Source	Pros	Cons	Challenges to Address	Wearable Applications	Implantable Applications
Neuro-transmitters (e.g., Acetylcholine, Dopamine)	- Present in synaptic regions and body fluids - High electrochemical activity	- Low concentrations in body fluids - Requires sensitive detection systems	- Enhancing catalytic efficiency - Improving enzyme selectivity and stability for neurotransmitters	- Used in wearable neurochemical monitoring - Power budget: ~4–30 µW	- Applicable for neurological implants - Power budget: ~1–10 µW
Glucose	- High availability in body fluids - Established enzymatic biofuel cell technology	- Enzyme instability over time - Dependence on glucose concentration in body fluids	- Improving enzyme stability - Scaling for long-term use - Miniaturization to maximize output per unit area	- Feasible for continuous glucose monitoring systems - Power budget: ~10–200 µW	- Suitable for low-power neuroprosthetics and diabetes implantable management systems - Power budget: ~10–500 µW
Lactate	- Abundant in sweat - Reliable bioenergy source for exercise-related applications	- Limited in resting conditions - Requires optimization of lactate oxidation	- Enzyme optimization for non-exercise states - Miniaturization for wearable platforms	- Effective for fitness wearables - Power budget: ~50–250 µW	- Less feasible due to fluctuating levels in vivo - Power budget: ~10–50 µW
Ethanol	- High energy density - Stable enzymatic activity	- Not naturally available in body fluids - Requires external input of ethanol	- Adapting ethanol systems for biomedical use - Developing safe ethanol reservoirs	- Limited applications due to external ethanol supply - Power budget: ~50–200 µW	- Feasible for specialized devices with ethanol reservoirs - Power budget: ~10–50 µW

## List of Abbreviations

**Abbreviation**	**Definition**
EBFCs	Enzymatic biofuel cells
BFCs	Biological fuel cells
SPBs	Self-powered biosensors
DET	Direct Electron transfer
IET	Indirect electron transfer
SAMs	self-assembled monolayers
EDC	1-ethyl-3(3-dimethylaminopropyl) carbodiimide
CNT	Carbon nanotube
GOx	Glucose oxidase
BOx	Bilirubin oxidase
GDH	Glucose dehydrogenase
MWCNTs	Multiwalled carbon nanotubes
SWNTs	Single-walled carbon nanotubes
MOF	Metal–organic framework
CNDs	Carbon nanodots
BP	Bucky paper
GO	graphene oxide
rGO	Reduced graphene oxide
ABTS	3-Aminopropyltriethoxysilane
BOD	Bilirubin oxidase
4-APA	4-amino phthalic acid
CS	Chitosan
CNCs	carbon nanochips
GCE	Glassy carbon electrode
Pt	Platinum
FRT	ferritin
AgNPs	Silver nanoparticles
ZnO	Zinc oxide
NADH	nicotinamide adenine dinucleotide with hydrogen
NG	Nitrogen-doped graphene
AuNPs	Gold nanoparticles
FDH	Formate dehydrogenase
PPCA	Poly(pyrrole-2-carboxylic acid
CPs	Conducting polymers
NGQDs	N-doped graphene quantum dots
PANI	Polyaniline
PB	Prussian blue
EPD	Bottom-up electrophoretic deposition
FTIR	Fourier-transform infrared spectroscopy
HRP	Horseradish peroxidase
SEM	Scanning electron microscopy
PQQ	pyrroloquinoline quinone
NQ	4-naphthoquinone
LOx	Lactate oxidase
PSS	Polystyrene sulfonate
AOx	Alcohol oxidase
ADH	Alcohol dehydrogenase
PVP	Polyvinylpyrrolidone
GBFC	Glucose Biofuel Cell
PPO	Polyphenol oxidase
ABS	Animal Brain Stimulator
